# Oocyte metabolic function, lipid composition, and developmental potential are altered by diet in older mares

**DOI:** 10.1530/REP-21-0351

**Published:** 2022-01-28

**Authors:** Giovana D Catandi, Lance LiPuma, Yusra M Obeidat, Lisa J Maclellan, Corey D Broeckling, Tom Chen, Adam J Chicco, Elaine M Carnevale

**Affiliations:** 1 Equine Reproduction Laboratory, Department of Biomedical Sciences, Colorado State University, Fort Collins, Colorado, USA; 2 Department of Biomedical Sciences, Colorado State University, Fort Collins, Colorado, USA; 3 Electronic Engineering Department, Hijjawi Faculty for Engineering Technology, Yarmouk University, Irbid, Jordan; 4 Proteomics and Metabolomics Facility, Colorado State University, Fort Collins, Colorado, USA; 5 Department of Electrical and Computer Engineering, Colorado State University, Fort Collins, Colorado, USA; 6 School of Biomedical Engineering, Colorado State University, Fort Collins, Colorado, USA

## Abstract

Dietary supplementation is the most feasible method to improve oocyte function and developmental potential *in vivo*. During three experiments, oocytes were collected from maturing, dominant follicles of older mares to determine whether short-term dietary supplements can alter oocyte metabolic function, lipid composition, and developmental potential. Over approximately 8 weeks, control mares were fed hay (CON) or hay and grain products (COB). Treated mares received supplements designed for equine wellness and gastrointestinal health, flaxseed oil, and a proprietary blend of fatty acid and antioxidant support (reproductive support supplement (RSS)) intended to increase antioxidant activity and lipid oxidation. RSS was modified for individual experiments with additional antioxidants or altered concentrations of n-3 to n-6 fatty acids. Oocytes from mares supplemented with RSS when compared to COB had higher basal oxygen consumption, indicative of higher aerobic metabolism, and proportionately more aerobic to anaerobic metabolism. In the second experiment, oocytes collected from the same mares prior to (CON) and after approximately 8 weeks of RSS supplementation had significantly reduced oocyte lipid abundance. In the final experiment, COB was compared to RSS supplementation, including RSS modified to proportionately reduce n-3 fatty acids and increase n-6 fatty acids. The ability of sperm-injected oocytes to develop into blastocysts was higher for RSS, regardless of fatty acid content, than for COB. We demonstrated that short-term diet supplementation can directly affect oocyte function in older mares, resulting in oocytes with increased metabolic activity, reduced lipid content, and increased developmental potential.

## Introduction

Oocyte viability is essential for female fertility. Maternal factors can affect oocyte quality, potentially by causing alterations in oocyte metabolism (Babayev & Seli 2015). Substrate preferences, lipid content, and metabolism vary among species, with oocyte lipid content affecting its reliance on the oxidation of carbohydrates or fatty acids (Dunning *et al.* 2014, Dalbies-Tran *et al.* 2020). Energy needed for oocyte development and maturation is primarily produced through aerobic mitochondrial metabolism (Ben-Meir *et al.* 2015, Cecchino *et al.* 2018). Mitochondria do not replicate until after blastocyst formation in several species, including the horse and human; therefore, mitochondria within the oocyte are responsible for providing energy during early embryo development (May-Panloup *et al.* 2007, Spikings *et al.* 2007, Wai *et al.* 2010, Hashimoto *et al.* 2017, Hendriks *et al.* 2019).

After the initiation of antral formation, approximately 2 months are required for the growth of the human oocyte and the development of the follicle to the ovulatory stage (Williams & Erickson 2012). The timeframe in the mare is likely similar, although not documented. Our ability to improve oocyte quality *in vivo* is limited. However, specific treatments or nutraceuticals can be used to target the follicle and oocyte during this growth phase. Although studies have reported some success with dietary supplementation (Nehra *et al.* 2012, Ben-Meir *et al.* 2015), no specific recommendations are available for women undergoing assisted reproductive technology (ART) procedures (Cecchino *et al.* 2018, Gaskins & Chavarro 2018), and less guidance is available for mares. Distribution of nutrients to the oocyte is complicated by dependence on the surrounding follicle (Richani *et al.* 2021). Granulosa cells line the ovarian follicle and have an essential role in metabolism and transport of nutrients from systemic circulation to follicular fluid, providing a local environment for the developing oocyte (Siu & Cheng 2013). Cumulus cells, which surround the oocyte, acquire and channel nutrients to the oocyte through cellular projections (Richani *et al.* 2021), convert energy forms for the oocyte, and potentially protect the oocyte from high levels of lipids (Aardema *et al.* 2013, Lolicato *et al.* 2015). Granulosa and cumulus cell metabolism is directly associated with oocyte metabolism (Cecchino *et al.* 2018). In women, granulosa cell oxidative stress is related to impaired oocyte quality and developmental potential (Jančar *et al.* 2007, Karuputhula *et al.* 2013). The development of microsensors that can measure real-time fluctuations in oxygen and pH allows us the ability to deduce single-oocyte aerobic and anaerobic metabolism and quantify them as oxygen consumption rate (OCR) and extracellular acidification rate (ECAR), respectively (Obeidat *et al.* 2018, 2019). Utilizing this technology, we recently confirmed less-efficient aerobic and anaerobic metabolism in oocytes collected from the dominant, maturing follicles of old compared to young mares, and impaired oocyte metabolism was associated with a significant reduction in developmental potential (Catandi *et al.* 2019, 2021). The effect of diet on the metabolism of individual oocytes has not been studied.

Dietary supplementation of antioxidants and polyunsaturated fatty acids (PUFAs), specifically long-chain omega-3 (n-3) fatty acids (FAs), has been studied in some species; but minimal information is available for the horse. Dietary antioxidants could counteract the effects of oxidative stress, and they have been associated with improved fertility in mice (Ben-Meir *et al.* 2015, Meldrum *et al.* 2016). The positive and negative effects of dietary n-3 PUFAs on female reproductive outcomes have been disputed (Gaskins & Chavarro 2018, Zarezadeh *et al.* 2019). The simplest primary form of dietary n-3 PUFA found in vegetable oils is α-linolenic acid (C18:3), which is an essential FA that can be converted to other long-chain, n-3 PUFAs including eicosapentaenoic acid (C20:5) and docosahexaenoic acid (DHA; C22:6) through desaturation and elongation reactions (Das 2006). However, these reactions can be inefficient with competitive inhibition of the rate-limiting enzymes, delta-6-desaturases; this can occur with high dietary omega-6 (n-6) PUFAs, such as linoleic acid (C18:2) (Zarezadeh *et al.* 2019). The natural equine diet is based on grazing and is composed of approximately 83% n-3 PUFA and 17% n-6 PUFA present in oils derived from leaves (Hallebeek & Beynen 2002, Frape 2004). Such PUFA composition is similar to the Mediterranean human diet, which is characterized by a higher ratio of n-3 to n-6 PUFA, with the ingestion of less carbohydrate-rich foods and more fruits and vegetables (de Lorgeril & Salen 2012, Broughton & Moley 2017), and improved embryo quality for patients undergoing ART procedures (Kermack *et al.* 2020). The modern equine diet commonly includes grain supplementation. Feeding a hay-based diet with 3 kg of cereal-based concentrate results in an inversion of FA content to approximately 5% n-3 PUFAs and 95% n-6 PUFAs (Hallebeek & Beynen 2002). These findings are consistent with the Western human diet characterized by high intake of cereal grains and more n-6 relative to n-3 PUFAs (Nehra *et al.* 2012, Hess & Ross-Jones 2014, Dhungana *et al.* 2016). The extent that absolute vs relative dietary content of n-3 and n-6 PUFAs affects fertility is yet to be determined. Diets considered ‘healthy’ (high consumption of vegetables, fruits, nuts, and meat) vs ‘unhealthy’ (high intake of solid oil, processed and junk food) resulted in the recovery of more metaphase II oocytes and higher chances of pregnancy after ART in women (Jahangirifar *et al.* 2019). Diet effects are likely systemic and multifactorial, with overall diet or complex supplementation having a greater impact on reproductive outcomes than individual nutrients.

In the present study, we used older mares to examine the hypothesis that dietary supplements fed for a limited time would affect oocyte metabolic function, lipid composition, and developmental potential. More specifically, we determined whether supplementation of compounds designed to promote overall wellness and cellular health would alter the follicle and oocyte destined for ovulation. We further elucidated the extent that altering antioxidants or n-3 vs n-6 PUFA in supplements would affect the oocyte.

## Materials and methods

### Animals, diet supplementation, and experimental designs

Colorado State University’s Institutional Animal Care and Use Committee approved all the procedures performed in this study. Three experiments were performed with similar groups of non-lactating mares of light-horse breeds during three consecutive breeding seasons. Some mares were used during multiple seasons. For all experiments, groups of non-lactating mares were housed in adjacent dry lots with sheds, mineral blocks, and water *ad libitum*; grass/alfalfa mix hay obtained from the same source in different years was fed at approximately 2% body weight (BW) daily. Nutritional and mycotoxin analyses of representative hay samples were performed and resulted in 14% crude protein, 1.8% crude fat, 14.5% crude fiber and were mycotoxin free. Diet additions were fed daily in the morning in individual pens to assure consumption.

Dietary supplements for the experiments were obtained from the same source (Platinum Performance, Inc., Buellton, CA, USA). All treatment groups received supplements designed to support equine wellness and gastrointestinal health (GI, Platinum Performance^®^GI (147 g), a combination of vitamins, trace minerals, amino acids, antioxidants, n-3 PUFA, probiotics, and prebiotics) and a proprietary blend of fatty acid and antioxidant support (reproductive support supplement, RSS) that was modified for individual experiments (Table 1). Grain and pelleted feed were manufactured by one source (Nutrena^®^, Cargill, Inc., Minneapolis, MN, USA) and were purchased from local sources.

**Table 1:** Experiments 1–3 with groups, dietary components, daily amount fed to mares, and basic experimental designs.

Experimental groups	Dietary component	Amount	Design
Exp 1: metabolic function			Two groups of mares were fed a control diet (COB) or supplemented diet (RSS1) for approximately 8 weeks prior to sample collection.
COB	Corn, oat, barley blend^a^	450 g	
	Corn oil	60 mL	
RSS1	GI supplement^b^	147 g	
	Flaxseed oil^c^	60 mL	
	Repro support supplement^d^	25.5 g	
	Pterostilbene	500 mg	
	Coenzyme Q10	500 mg	
	Pyrroloquinoline quinone	40 mg	
Exp 2: lipid profiles			Samples were collected from single group of mares prior to any diet supplementation (control, CON) and after 8 weeks of feeding RSS2.
CON	Pretreatment sample	0	
RSS2^g^	GI supplement	147 g	
	Flaxseed oil	60 mL	
	Repro support supplement	51 g	
	SafeChoice (pelleted feed)	680 g	
	Grain mix with molasses	227 g	
Contemporary samples			Contemporary samples to Exp 2 were collected from four mares prior to and after 8 weeks of CoQ. Samples were assessed for an effect of time; no direct comparisons were made.
CONQ	Pretreatment sample	0	
CoQ^g^	Coenzyme Q10	500 mg	
Exp 3^f^: developmental potential			Three groups of mares were fed a control diet (COB), RSS3 (same as RSS2), or RSS3M in which RSS3 was modified by reducing n -3 fatty acids and increasing n -6 fatty acids.
COB	Same as COB, Exp 1		
RSS3	Same as RSS2 without pelleted feed and grain mix		
RSS3M	GI supplement	147 g	
	Corn oil	60 mL	
	Repro support supplement without DHA^e^	50 g	

^a^Provided 5.04 g linoleic acid (LA) and 0.19 g alpha-linolenic acid (ALA); ^b^Platinum Performance^®^ GI, Platinum Performance Inc., Buellton, CA; ^c^Healthy Weight, Platinum Performance Inc. provided 8.46 g LA, 30.8 g ALA, and 480 IU d-alpha-tocopherol acetate; ^d^Reproductive support supplement proprietary blend, Platinum Performance Inc. provided ALA, docosahexaenoic acid (DHA), ascorbic acid, acetyl-l-carnitine, l-carnitine tartrate, and d-alpha-tocopherol acetate; ^e^Proprietary blend, Platinum Performance Inc. provided ALA, ascorbic acid, acetyl-l-carnitine, l-carnitine tartrate, and d-alpha-tocopherol acetate; ^f^Oocytes collected during Experiment 1 (COB and RSS1) were also used to assess oocyte developmental potential in Experiment 3; ^g^Post-treatment sample.

**Figure 1:**
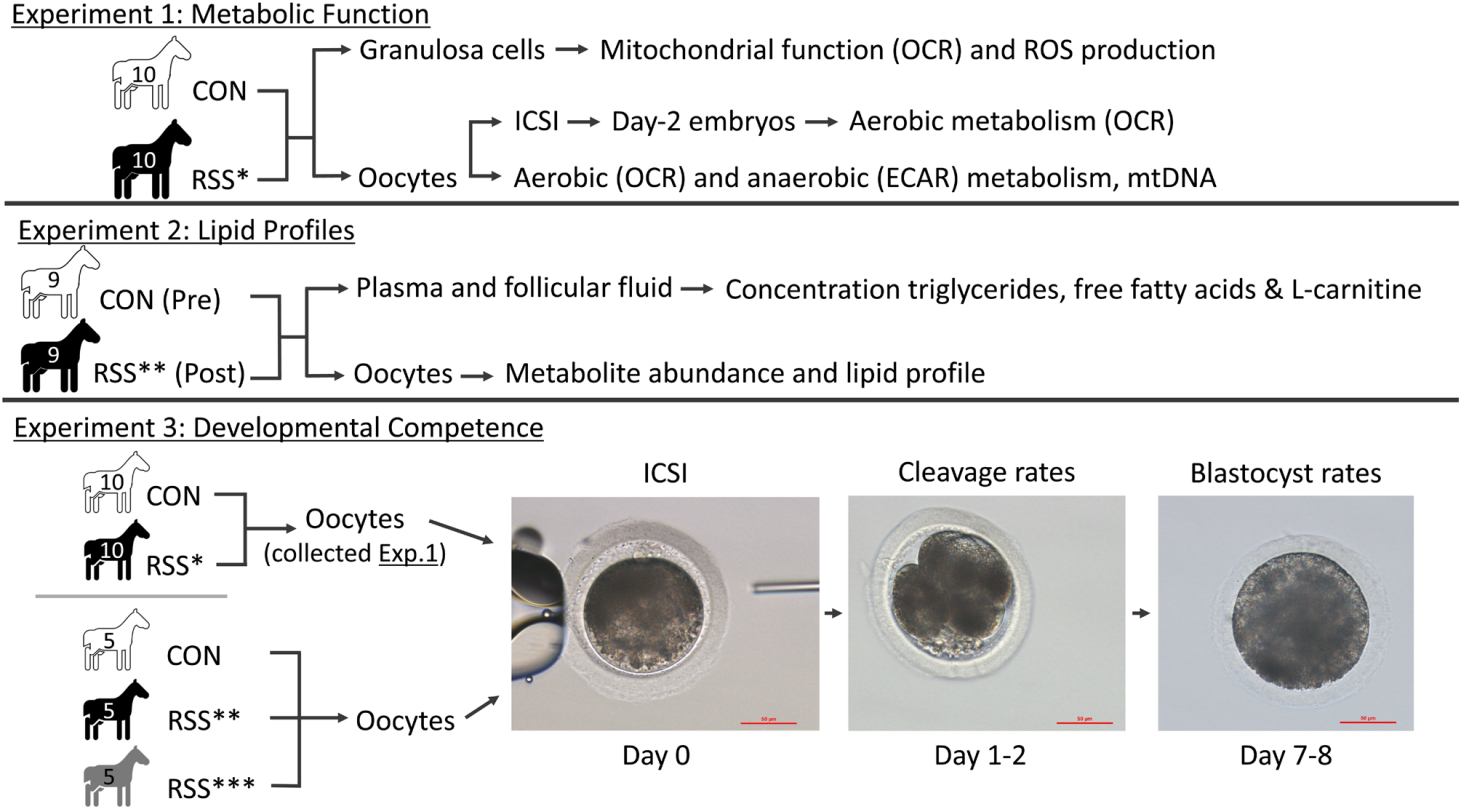
General experimental design. Control (CON) diets included only hay (Experiment 2) or hay and grain products (Experiments 1 and 3). Reproductive support supplements (RSS*, RSS**, and RSS***) differed as described in Table 1. Samples were collected after ≥8 weeks of supplementation of matched groups of mares in Experiments 1 and 3. In Experiment 2, samples were collected from the same mares pre- (CON) and post-supplementation (RSS**). Representative images of an oocyte, early embryo (day 2), and blastocyst are shown from a treatment mare (RSS**). The number of mares per group is shown within the horse icons.

Three experiments were performed to evaluate the effect of feeding supplements to support reproduction in older mares, with some variation in treatment groups to test different types of compounds and reproductive endpoints. In the first experiment, oocyte metabolic function was assessed after feeding dietary supplement with additional antioxidants. In the second experiment, oocyte lipid profiles were assessed before and after feeding the diet supplement. In the third experiment, oocyte developmental potential was compared after supplementation with and without altering the relative abundance of omega-3 vs omega-6 PUFAs. The experimental design for each experiment is summarized in Fig. 1.

In Experiment 1 (metabolic function), mares were provided group-specific feeding regimes for 8–13 weeks before samples were collected in July and August to assess granulosa cell, oocyte, and early embryo metabolic function. Twenty mares between 13 and 23 years (mean age of 18.5 years) and 485 and 670 kg BW were paired by age and body type. One mare from each pair was randomly assigned to one of two groups, with the other mare assigned to the remaining group. Although each group contained one 13-year-old mare, the remaining mares were older (≥17 years). The control group (COB, mean age of 18.6 years) received 450 g of a mix of corn, oats, and barley (Nutrena^®^ C.O.B.) topped with 60 mL of corn oil (Mazola^®^, ACH Food Companies, Inc., Memphis, TN, USA), representing approximately 10% of the daily caloric intake. The relative n-6 to n-3 PUFA ratio of the COB supplementations was approximately 42:1. The treatment group of mares (RSS1, mean age of 18.5 years) was fed approximately equicaloric commercial supplements including GI and RSS at 25.5 g daily (Table 1). In addition, flaxseed and natural vitamin E (d-alpha-tocopherol acetate) oil (healthy weight oil (60 mL)) supplied additional n-3 PUFA, providing a relative n-6 to n-3 PUFA ratio of 0.3:1. Specific antioxidants (coenzyme Q10 (CoQ), 500 mg; pterostilbene, 500 mg; pyrroloquinoline quinone, 40 mg) were also provided to the treatment group (RSS1, Table 1).

For Experiment 2 (lipid profiles), oocytes, follicular fluid, and blood were collected from mares in May or June (pre-treatment). The mares were then fed supplements for 8–10 weeks before post-treatment samples from the same mares were collected in August. Oocyte lipid composition in addition to follicular and systemic concentrations of lipids and l-carnitine was determined pre- and post-supplementation from nine mares (16–22 years, mean age of 18.7 years, and 472 and 577 kg BW). For this experiment, mares were supplemented daily with GI, RSS (51 g), and 60 mL of flaxseed oil (RSS2, Table 1). The supplements were mixed with a pelleted complete feed (Nutrena^®^ SafeChoice Original, 14% crude protein, 7% crude fat, 680 g) and a mixed grain blend with molasses (Nutrena^®^ Rocky Mountain Sweet Feed, 8% protein and 2% fat, 227 g) to increase palatability.

During the same period of time, a contemporary group of four mares (16–22 years, mean age of 18.7 years, 453–568 kg BW) were maintained on the same hay diet and in the same housing conditions as mares in Experiment 2, but they were supplemented only with an antioxidant (CoQ, 500 mg daily) and no other dietary additives (Table 1). Oocyte metabolite content was also assessed for these mares to determine whether oocyte composition was altered over time in mares that were not given the RSS. No direct statistical comparisons were made between the treated mares in Experiment 2 and in this contemporary group of mares.

For Experiment 3 (developmental competence), oocytes were obtained from the mares in Experiment 1 or were obtained from additional groups of mares fed varying proportions of n-3 and n-6 PUFAs. For these mares, supplements were provided for 8–17 weeks before oocytes were collected and injected with sperm to assess developmental potential. Fifteen mares aged 18–24 years (mean age of 20.6 years, 440–610 kg BW) were grouped by age and then randomly assigned to one of three groups. The control group of mares (COB, *n* = 5, mean age of 20.4 years) received corn, oats, and barley, and corn oil supplements (Table 1). The second group of mares (*n*= 5, mean age of 20.4 years) received RSS3 (same supplements as RSS2 in Experiment 2), with a relative n-6 to n-3 PUFA ratio of 0.3:1. For the third group of mares (RSS3M, *n *= 5, mean age of 20.8 years), the supplements were modified by removing DHA from RSS and flaxseed oil was replaced with corn oil, reducing n-3 PUFAs and increasing n-6 PUFAs in the diet to n-6 to n-3 PUFA ratio of 2.1:1 (Table 1).

### Oocyte collection and maturation

Oocytes were collected from dominant, follicular-phase follicles to provide consistency in the stage of development and evaluation of oocytes which were destined to ovulate naturally. Follicular maturation was induced during the follicular phase when a dominant follicle ≥35 mm in diameter and endometrial edema were observed using ultrasonography. Induction occurred from the administration of human chorionic gonadotropin (2000 IU, i.v.; Chorulon, Merck Animal Health, Madison, NJ, USA) and deslorelin acetate in an aqueous base (0.75 mg, i.m.; Precision Pharmacy, Bakersfield, CA, USA) in Experiments 1 and 2 and histrelin in aqueous base (0.5 mg, i.m., Doc Lane, Lexington, KY, USA) in Experiment 3. Cumulus oocyte complexes (COCs) were collected by transvaginal, ultrasound-guided follicular aspiration of dominant follicles at 16 ± 2 h after induction in Experiment 1 and at 20 ± 2 h in Experiments 2 and 3 as described previously (Carnevale 2016). Recovered COCs were incubated in media (TCM199 with Earle’s salts (GibcoTM, Thermo Fisher Scientific) with 10% fetal bovine serum (FBS), 25 µg/mL of gentamicin, and 0.2 mM pyruvate) at 38.2°C in an atmosphere of 5% CO_2_ and air for 26 ± 2 h in Experiment 1 and 22 ± 2 h in Experiment 3. After maturation, oocytes were stripped of cumulus cells by sequential pipetting in a 3-(N-morpholino)propanesulfonic acid (MOPS)-buffered medium (G-MOPS, Vitrolife, Englewood, CO, USA) with 0.04% BSA (Sigma–Aldrich) and hyaluronidase (200 IU/mL; Sigma–Aldrich). For electrochemical measurements of basal and maximal OCR and ECAR in Experiment 1, oocytes were moved to a MOPS-buffered medium (G-MOPS) with 0.04% BSA at 38.2°C until the assay. In Experiment 2, recovered oocytes were stripped of cumulus cells as described above, carefully evaluated to confirm complete removal of cumulus cells, fixed in 100 μL of 50% methanol solution, snap-frozen in liquid nitrogen, and stored at −80°C until mass spectrometry analyses.

### Granulosa cell collection, OCR, and reactive oxygen species production assays

In Experiment 1, granulosa cells were collected at the time of oocyte recoveries. For mitochondrial OCR and ROS release data collection, granulosa cells (COB, *n* = 7 and RSS1, *n* = 5) were separated from the follicular aspirates, suspended in flush solution (Vigro Complete Flush Solution, Vetoquinol, Fort Worth, TX, USA), and pelleted for further use. The flush solution supernatant was aspirated, and granulosa cells were resuspended and washed in mitochondrial respiration medium (MiR05) containing 0.5 mM EGTA, 3 mM MgCl_2_, 60 mM lactobionic acid, 20 mM taurine, 10 mM KH_2_PO_4_, 20 mM HEPES, and 110 mM d-sucrose. Cells were resuspended and washed twice by pulling 1000 μL of cells from the flush solution and placing them in a 2-mL eppendorf tube containing 1000 μL of MiR05. Cells were then mechanically washed by pipetting up and down before being pelleted and washed again in a separate eppendorf tube containing 1000 μL of MiR05. The entire cell suspension was added to a 2-mL chamber in an Oxygraph-2k high-resolution respirometer (Oroboros Instruments, Innsbruck, Austria). OCR was monitored in real-time by resolving changes in the negative time derivative of the chamber oxygen concentration signal. This signal was normalized to the protein concentration of the granulosa cell pellet that was collected at the end of experiments. Respirometry chambers were maintained at 37°C under atmospheric oxygen concentration (100–200 µM O_2_). Basal OCR (respiration of intact cells supported by endogenous substrates) was measured prior to permeabilization of cell membrane with digitonin (15 µg/mL) to provide mitochondrial access to cell impermeable substrates. Mitochondrial respiratory flux and maximal OCR were stimulated by the addition of multiple substrates in the following sequence: 1 mM malate, 5 mM pyruvate, 2.5 mM ADP, 10 mM glutamate, and 10 mM succinate as previously described (Chicco *et al.* 2018). For the determination of ROS release, 10 µM of Amplex Red and horseradish peroxidase, at a final concentration of 1 U/mL, were added to the oxygraph chamber after the addition of granulosa cells for fluorescence measurement. Horseradish peroxidase combines with hydrogen peroxide (H_2_O_2_, a membrane-permeable ROS species) and irreversibly oxidizes Amplex Red to resorufin (Ex/Em 571/585 nm) while reducing H_2_O_2_ to two equivalents of H_2_O (Goo *et al.* 2013). ROS data are presented as the rate of release per second.

### *In vitro* embryo production

Frozen-thawed semen from a single ejaculate from one stallion was used for sperm injections. Approximately, one-tenth of a 0.25-mL straw of frozen semen was cut under liquid nitrogen and thawed directly in 1 mL of MOPS-buffered medium (G-MOPS) with 0.04% BSA at 38.2°C; one sperm was selected and prepared for sperm injection as previously described (Gonzalez-Castro & Carnevale 2018). Intracytoplasmic sperm injection (ICSI) was performed in a MOPS-buffered medium (G-MOPS) with 0.04% BSA using a micromanipulator (Narishige Group, Amityville, NY, USA) and a piezo-driven injection system (Prime Tech, Ibaraki, Japan). After sperm injection, presumptive zygotes were placed into an embryo culture medium (global^®^, LifeGlobal Group, Guilford, CT, USA) with 10% FBS in individual 30-μL droplets under paraffin oil (OVOIL™, Vitrolife) and incubated at 38.2°C in 5% O_2_, 7% CO_2_, and 88% N_2_.

In Experiment 1, 2 days after ICSI (46–57 h, mean of 52.2 h), early embryos with normal morphology and two to eight cells (mean of four cells) were used for the measurement of basal OCR (COB, *n* = 11 and RSS1, *n* = 12). A set of early embryos were stimulated for maximal OCR (COB, *n* = 6 and RSS1, *n* = 6). Additional embryos were cultured in Experiments 1 (COB, *n* = 13 and RSS1, *n* = 12) and 3 (COB, *n* = 19; RSS3, *n* = 15; and RSS3M, *n* = 24) to determine blastocyst development rates. These embryos were moved 5 days after ICSI into individual 30-µL droplets of a second culture medium (global^®^ for fertilization, LifeGlobal Group) with 10% FBS. Embryos were observed daily until blastocyst formation or degeneration (Carnevale & Metcalf 2019).

### Oocyte and early embryo OCR and ECAR assays

Metabolic analyses of oocytes (COB, *n* = 4 and RSS1, *n* = 4) and early embryos in Experiment 1 were performed using a microchamber with electrochemical-based oxygen and pH sensors as described in detail (Obeidat *et al.* 2019). Before each oocyte or early embryo assay, the microchamber was filled with 120 μL of MOPS-buffered medium (G-MOPS) with 0.04% BSA, overlaid with 120 μL of paraffin oil (OVOIL™) to limit the chamber from atmospheric oxygen diffusion. The three-electrode system for the oxygen sensor was connected to a potentiostat (Quadstat EA 164H, eDAQ Inc., Colorado Springs, CO, USA) that applied −0.6 V to the sensor and monitored the decrease in oxygen reduction current over time. The pH sensor was connected to a custom-made Ina333 instrumentation amplifier circuit that measured the change in voltage. The starting oxygen concentration and pH of the medium were measured as baseline current. After that, individual oocytes or early embryos were transferred into the microchamber.

Calculations of sample OCR and ECAR were based on the rates of change for oxygen and pH, respectively, from baseline values over time. Initially, basal OCR was assayed for 5 min, followed by basal ECAR assessment for 2 min. Three titrations of 1 µM of carbonyl cyanide *m*-chlorophenyl hydrazone (CCCP), a mitochondria uncoupler that stimulates maximal oxygen consumption, were performed for oocyte and early embryos, with OCR measurements for 5 min after each CCCP addition. Maximal OCR was defined as the highest observed value during CCCP titrations. An additional measurement for oocyte ECAR was determined after the first addition of CCCP, as an indication of the oocyte’s ability to use anaerobic pathways when mitochondrial energy production is limited. After metabolic readings, individual oocytes were stored at −80°C until mtDNA quantification.

### Oocyte mitochondrial DNA content absolute quantification

Mitochondrial DNA (mtDNA) content of single oocyte (COB, *n* = 16 and RSS1, *n* = 18) was quantified by real-time PCR (qPCR) as previously described (Pasquariello *et al.* 2019, Catandi *et al.* 2021). Kits and supplies from one source (Qiagen) were used unless noted. Briefly, DNA extraction of individual oocytes was performed using the QIAamp DNA micro kit according to the manufacturer’s protocol with the addition of carrier RNA (1 µg) to each sample. The DNA sample was eluted with 50 μL of buffer AE (supplied with the kit) and analyzed for qPCR using an absolute quantification assay. To this end, quantification standards were prepared; a 1096-bp fragment of the I-rRNA region of equine mtDNA was amplified by PCR using the LongRange PCR Kit and the primer pair 5’-AGCAATTTCGGTTGGGGTGA-3’ and 5’-GCTCGGTTGGTTTCGGCTAA-3’. The fragment was then purified using the Qiaquick PCR purification kit and cloned using the Qiagen PCR cloning kit. Plasmid DNA containing the amplified mtDNA fragment was purified from bacteria using the Qiaprep miniprep kit. A standard curve was generated by using seven 10-fold serial dilutions (10^7^ to 10 copies), and standard curve correlation coefficients were greater than 0.98. Real-time quantitative PCR using the primer pair 5’-ATGGTTTGTGCTACTGCTCG-3’ and 5’-GCCCTAACCCTGGCCTTAAC-3’ was run in triplicate for each standard dilution and sample in 10-μL reaction using PowerUp SYBR Green master mix (Applied Biosystems), a LightCycler 480II (Roche Applied Science). The program of amplification was set as follows: 50°−C for 2 min for the first cycle, 95°C for 2 min for the second cycle, 95°C for 15 s, and 60°C for 1 min for 40 cycles; a melting curve was run to assess the specificity of the primers. Samples and standard curve were run on the same plate. Copy numbers of mtDNA in each oocyte were generated from the equation of Ct value against copy number for the corresponding standard curve.

### Oocyte metabolomics analyses by liquid chromatography coupled to mass spectrometry

Metabolites in oocytes (RSS2, *n* = 9 pre- and post-supplementation) were first extracted by adding 400 μL of 100% liquid chromatography-mass spectrometry (LCMS)-grade methanol to each sample while still frozen. The samples were then shaken for 1 h at 4°C. The samples were sonicated in a cold bath for 30 min, shaken again for 1 h at 4°C, and sonicated in a cold bath for 30 min. The samples were centrifuged briefly, and all 500 μL of sample was dried completely with nitrogen and resuspended in 60 μL of 1:1 methanol/toluene. A quality control (QC) sample was pooled by taking 15 μL per sample.

Five microliters of extract were injected onto ACQUITY UPLC system (Waters, Milford, MA, USA) in randomized order with a QC injection after every six samples. Separation was achieved using ACQUITY UPLC CSH Phenyl Hexyl column (1.7 μM, 1.0 × 100 mm) (Waters), using a gradient from solvent A (water, 2 mM ammonium formate) to solvent B (acetonitrile, 0.1% formic acid). Injections were made in 99% A, held at 99% A for 1 min, ramped to 98% B over 12 min, held at 98% B for 3 min, and then returned to starting conditions over 0.05 min, and allowed to re-equilibrate for 3.95 min, with a 200 μL/min constant flow rate. The column and samples were held at 65°C and 6°C, respectively. The column eluent was infused into a Xevo G2-XS Q-TOF-MS (Waters) with an electrospray source in positive mode, scanning 50–1200 m/z at 0.2 s per scan, alternating between MS (6 V collision energy) and MSE mode (15–30 V ramp). Calibration was performed using sodium formate with 1 ppm mass accuracy. The capillary voltage was held at 700 V, source temperature at 140°C, and nitrogen desolvation temperature at 600°C with a desolvation gas flow rate of 1000 L/h.

Raw mass spectrometry data were processed using an R-based workflow for feature detection, retention time alignment, feature grouping, peak filling, and feature clustering. RAMClustR version 1.1.0 in R version 3.6.3 was used to normalize, filter, and group features into spectra. XCMS (Smith *et al.* 2006, Tautenhahn *et al.* 2008) output data were transferred to ramclustR object using the rc.get.xcms.data function. Feature data were extracted using the xcms featureValues function; features that failed to demonstrate signal intensity of at least three-fold greater in QC samples than in blanks were removed from the feature dataset (3642 of 24,810 features were removed). Features with missing values were replaced with small values simulating noise. For each feature, the minimum detected value was multiplied by 0.1. Noise was then added using a factor of 0.1. The absolute value was used to fill the noise values to ensure that only non-negative values were carried forward. Variance in quality control samples was described using the rc.qc function within ramclustR. Features were normalized by linear regression of run order vs qc feature intensities to account for instrument signal intensity drift. Only features with a regression *P* -values less than 0.05 and an r-squared greater than 0.1 were corrected. Features were additionally normalized to the total extracted ion signal to account for differences in total solute concentration. Normalized peak areas for individual metabolites were compared between pre- and post-supplementation paired samples; within each lipid category, individual metabolites were summed to obtain a peak area for the total normalized abundance for each lipid category.

### Follicular fluid and blood collection and analyses

In Experiment 2, plasma and follicular fluid were collected for triglyceride, fatty acid, and l-carnitine assays (RSS2, *n* = 9 RSS2 pre- and post-supplementation). To prevent cellular and blood contamination of follicular fluid samples, the aspiration needle was not rinsed with media and was inserted into the central antrum of the follicle before approximately 5 mL of follicular fluid were gently aspirated into a collection tube. The follicular fluid was aliquoted and stored at −80°C until assays. Blood was collected by jugular venipuncture prior to morning feeding and centrifuged at 200 ***g*** for 10 min at room temperature; supernatant was recentrifuged at 1500 ***g*** for 10 min at room temperature, then aliquoted, and stored at −80°C until assays.

Triglyceride concentrations in plasma and follicular fluid samples were determined using a colorimetric assay kit (Cayman Chemicals) according to kit instructions. The 96-well, non-treated microplate (Thermo Fisher Scientific) was read at 540-nm absorbance on a Synergy 2 microplate reader (Biotek). All samples were assayed on a single plate. The intra-assay coefficient of variation was 1.35%, and the minimal detectable concentration was 1 mg/dL. Concentrations of free fatty acids in plasma and follicular fluid were determined with a colorimetric assay kit (Bioassay Systems, Hayward, CA) according to the manufacturer’s instructions. The 96-well microplate was read at 570-nm absorbance using the same microplate reader. The intra-assay coefficient of variation was 3.84%, and the sensitivity was 7 µM. l-Carnitine concentrations in plasma and follicular fluid were determined with a colorimetric assay kit (BioVision) following the kit instructions. The 96-well microplate was read at 570-nm absorbance with the same equipment. The intra-assay coefficient of variation was 2.14%, and the limit of detection was 10 µM.

### Statistical analysis

Statistical analyses were completed using GraphPad Prism 8.0.2 (GraphPad Software, Inc.). Student’s *t-*tests were used to compare continuous data in Experiment 1 and Fisher’s exact tests and chi-square tests were used to compare cleavage and blastocyst rates in Experiments 1 and 3. In Experiment 2, ANOVA with Kenward–Roger degrees of freedom and false discovery rate adjustments was used to compare metabolite abundance in pre- and post-supplementation oocytes; paired *t*-tests and Wilcoxon tests were used to compare continuous data. Values of *P* < 0.05 were considered significant, and *P* ≤ 0.1 was considered trending toward significance. Results are presented as mean ± s.e.m.

## Results

### Experiment 1: effect of diet supplementation on cell metabolic function

Mares were supplemented with grain (COB, corn, oats, and barley) or RSS with additional antioxidants (RSS1) prior to the assessment of aerobic metabolism, based on OCR, or anaerobic metabolism, based on ECAR, of granulosa cells, oocytes, and early cleavage embryos.

**Figure 2:**
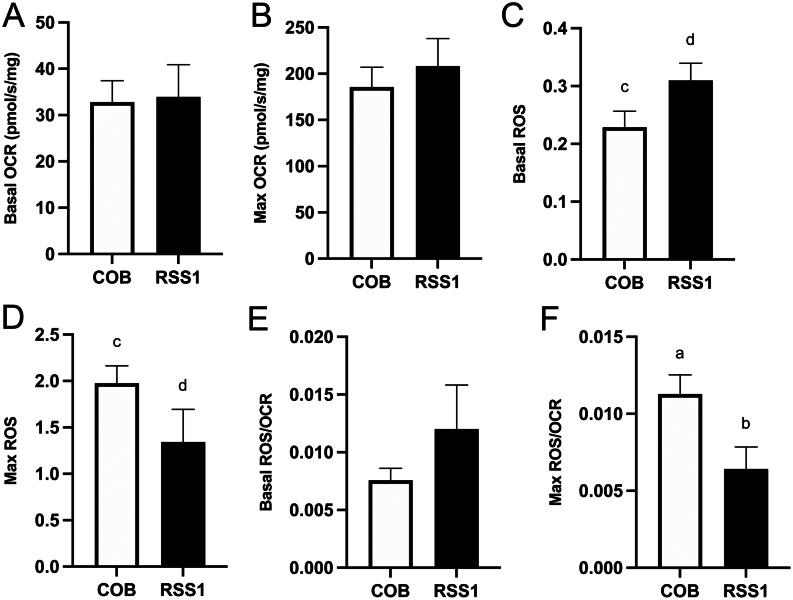
Granulosa cell aerobic metabolism, based on oxygen consumption rate (OCR), and production of reactive oxygen species (ROS) under basal and stimulated conditions from older mares supplemented with grain and corn oil (COB) or complex nutrients to support health and reproductive function (RSS1). (A) Basal OCR, (B) maximal (stimulated) OCR, (C) ROS formation under basal conditions, (D) ROS formation under stimulated conditions, (E) proportion of basal OCR related to ROS production, and (F) proportion of maximal OCR related to ROS production (COB, *n* = 7; RSS1, *n* = 5). Barcharts present means ± s.e.m. Different superscripts indicate differences (a,b;, *P*< 0.05) or a tendency to differ (c,d; *P*≤ 0.1). Supplement components are listed in Table 1.

Supplementations did not significantly affect granulosa cell aerobic metabolism or ROS production (Fig. 2A, B, C, D and  E). However, the production of ROS relative to aerobic metabolism (ROS/OCR) was higher (*P*= 0.03) for mares fed grain than the RSS under substrate-stimulated respiration, indicating that a higher proportion of oxygen consumption resulted in ROS production than in the generation of ATP (Fig. 2F).

**Figure 3:**
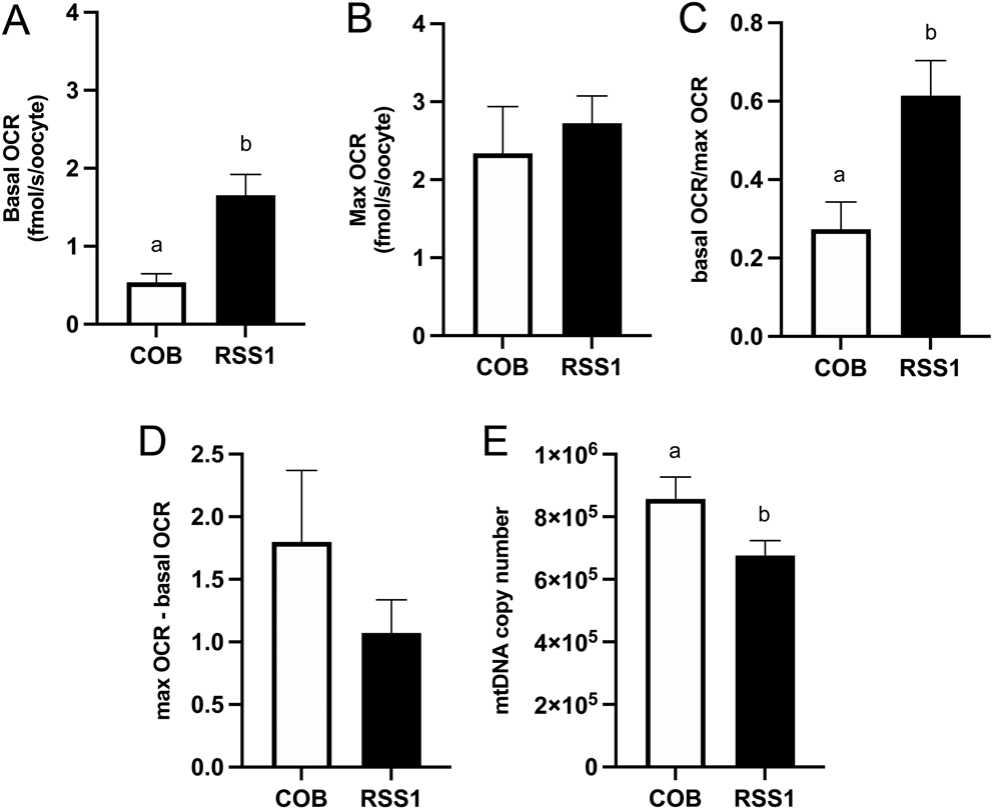
Aerobic metabolism, based on oxygen consumption rate (OCR), and DNA copy numbers in metaphase II oocytes from older mares supplemented with grain and corn oil (COB) or complex nutrients to support health and reproductive function (RSS1). (A) Basal OCR, (B) maximal OCR, (C) mitochondrial efficiency (basal OCR/max OCR), (D) mitochondrial reserve capacity (max OCR–basal OCR) (COB, *n* = 4; RSS1, *n* = 4), and (E) quantification of mtDNA copy number (COB, *n* = 16; RSS1, *n* = 18). Barcharts present means ± s.e.m. Different superscripts indicate differences at *P*< 0.05.

**Figure 4:**
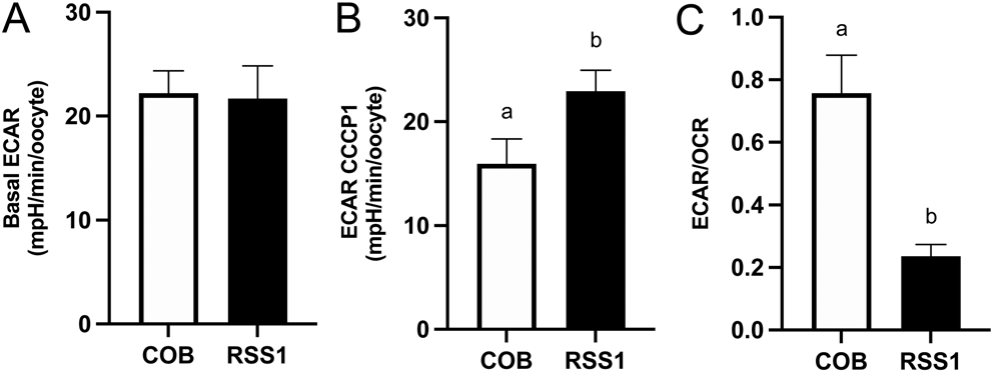
Anaerobic metabolism, based on extracellular acidification rate (ECAR), of metaphase II oocytes from older mares supplemented with grain and corn oil (COB) or complex nutrients to support health and reproductive function (RSS1). (A) Basal ECAR, (B) ECAR after first addition of CCCP (COB, *n* = 9; RSS1, *n* = 8), and (C) proportion of basal anaerobic to aerobic metabolism (COB, *n* = 4; RSS1, *n* = 4). Barcharts present mean ± s.e.m. Different superscripts indicate differences at *P*< 0.05.

Basal, but not maximal, aerobic metabolism was lower (*P*= 0.008) for oocytes collected from mares fed grain than the RSS (Fig. 3A and  B). Mitochondrial efficiency, representing the proportion of maximal cell respiratory capacity used during basal metabolism (basal OCR/maximal OCR), was lower (*P*= 0.02) when mares were fed grain than the diet support supplement (Fig. 3C). However, mitochondrial reserve capacity (maximal OCR–basal OCR) was similar (*P*= 0.3) between the groups, suggesting that the oocytes were capable of similar responses to energy demands (Fig. 3D). As aerobic respiration occurs in mitochondria, mitochondria DNA copy numbers were analyzed as an indicator of the number of mitochondria within oocytes. However, in contrast to metabolic activity, mtDNA were higher (*P*= 0.04) in oocytes from mares fed grain than the RSS (Fig. 3E). Oocyte basal anaerobic metabolism, based on the ECAR, did not differ between groups (Fig. 4A); although when stimulated, maximal anaerobic metabolism was higher (*P*= 0.04) for oocytes from mares fed the support supplement when compared to grains (Fig. 4B). Oocytes from mares fed grains used proportionately more (*P*= 0.006) anaerobic to aerobic metabolism (based on ratios of ECAR/OCR and reflecting the glycolytic rate to oxidative phosphorylation rate) (Fig. 4C). No significant differences were observed for the metabolic activity of early embryos resulting from ICSI of oocytes from mares supplemented with grains or RSS (Supplementary Fig. 1A and B, see section on supplementary materials given at the end of this article). In total, the results of the first experiment demonstrate differences in mitochondrial function in the oocytes from old mares fed grain products or provided nutrients for reproductive support.

### Experiment 2: effects of diet supplement on systemic, follicular, and oocyte lipid concentrations

**Figure 5:**
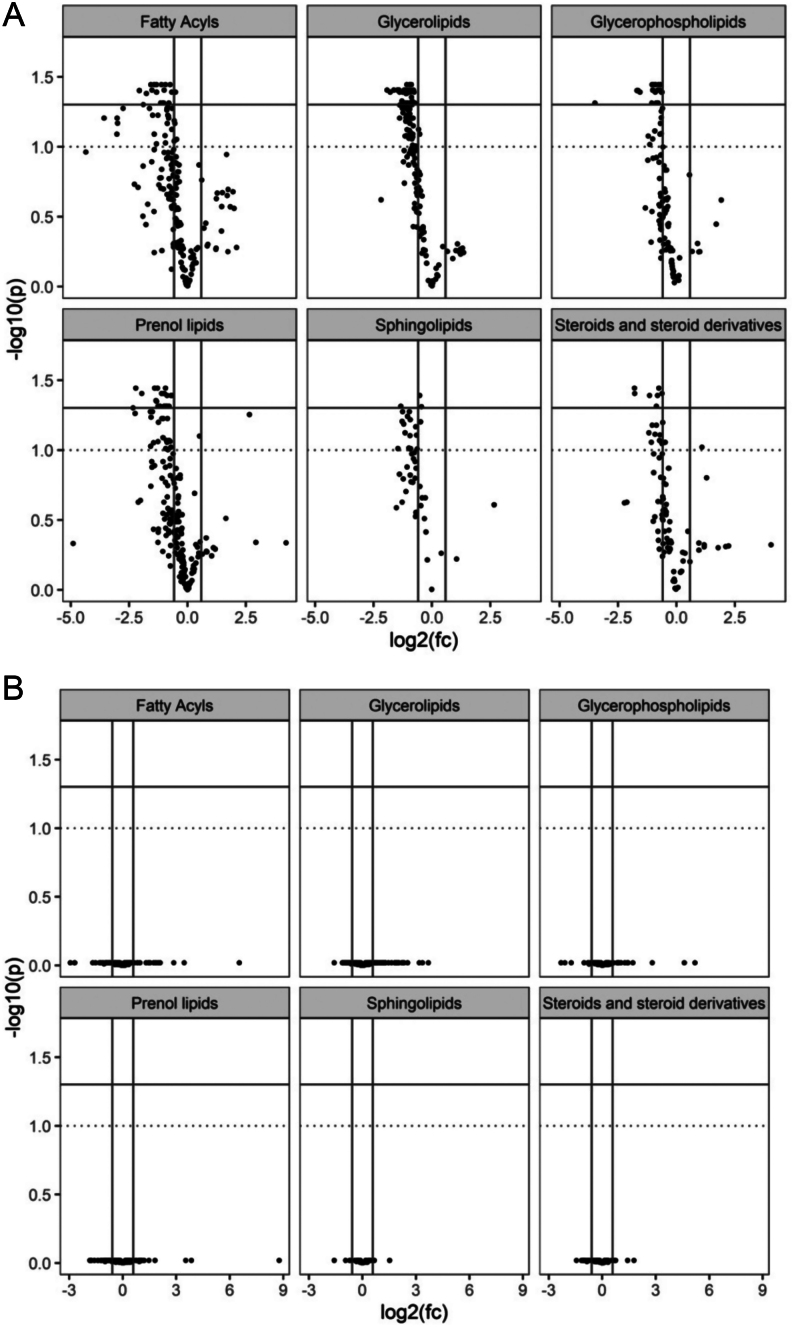
Volcano plots illustrating lipid categories in oocytes from older mares (A) pre- and post-supplementation with complex nutrients to support health and reproductive function (RSS2, *n* = 9) and (B) pre- and post-supplementation with coenzyme Q10 (*n*= 4). Negative log_2_(fc) indicates lipids that were reduced in oocytes post-diet supplementation, while positive log_2_(fc) indicates lipids that were elevated in post-diet supplementation oocytes. The horizontal bars indicate significance at *P*< 0.05 (solid line) and *P*< 0.1 (dotted line).

**Figure 6:**
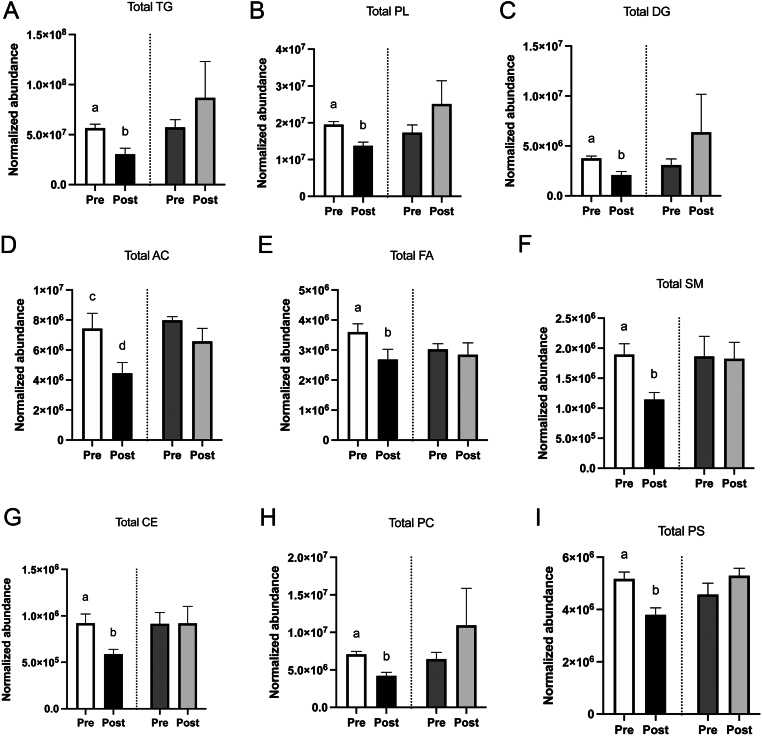
Normalized abundance of total (A) triglycerides, (B) glycerophospholipids, (C) diacylglycerols, (D) acylcarnitines, (E) free fatty acids, (F) sphingomyelins, (G) cholesteryl esters, (H) glycerophosphocholines, and (I) glycerophosphoserines in oocytes from older mares pre- (white bars) and post-supplementation with RSS2 (black bars; *n* = 9) and pre- (dark gray bars) and post-supplementation with coenzyme Q10 (light gray bars; *n* = 4). Barcharts represent means ± s.e.m. Different superscripts for pre- and post-supplementation with RSS2 indicate differences (a,b; *P*< 0.05 or c,d; *P*< 0.1). No significant differences were observed pre- and post-supplementation with coenzyme Q10.

Oocyte metabolome and systemic and follicular concentrations of lipids were assessed for mares prior to and after being fed RSS2. A total of 1585 metabolites were assessed in oocytes; 441 metabolites differed (*n*= 211, *P*  < 0.05) or tended to differ (*n*= 230, *P* ≤ 0.1) in oocytes that were collected before when compared to after diet supplementation with most differences observed in lipids (*n*= 802 total lipid metabolites, *n* = 118, *P*  < 0.05 and *n* = 139, *P* ≤ 0.1). Differences in lipid abundance were primarily noted for glycerolipids (Fig. 5A), although generally species of lipids were consistently less abundant in oocytes collected after than before diet supplementation. Normalized abundance of total triglycerides, glycerophospholipids, diacylglycerols, free fatty acids, sphingomyelins, cholesteryl esters, glycerophosphocholines, and glycerophosphoserines were significantly higher before when compared to after supplementation (Fig. 6). In a contemporary group of mares consuming only CoQ in addition to hay, lipid content and normalized abundance of lipid categories did not differ before or after feeding the antioxidant (CoQ) during the same time interval (Figs 5B and  6). Results from this contemporary group of mares demonstrate that oocyte lipid content did not change over time when the RSS was not fed. Therefore, dietary supplementations resulted in changes in oocyte composition, most notably in lipid abundance. Oocyte metabolites affected by the diet supplements are presented in Supplementary Tables 1 and 2.

**Figure 7:**
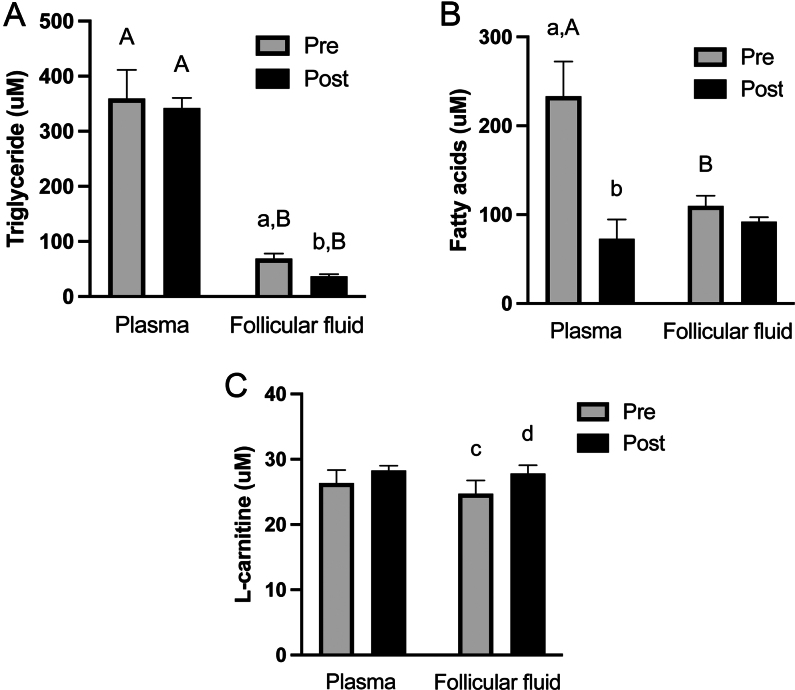
Concentrations of (A) triglycerides, (B) free fatty acids, and (C) l-carnitine in plasma and follicular fluid from older mares (*n*= 9) pre- and post-supplementation with complex nutrients to support health and reproductive function (RSS2). Barcharts represent means ± s.e.m. Different superscripts between pre- and post-supplementation for plasma or for follicular fluid represent differences (a,b; *P*< 0.05 and c,d; *P*< 0.1); different superscripts for the same endpoint between plasma and follicular fluid indicate significance (A,B; *P*< 0.05).

Systemic and follicular fluid concentrations of lipids were assessed before and after feeding the diet supplement. Control (pre-supplement) concentrations of triglycerides and free fatty acids were higher in plasma than in follicular fluid. Diet supplementation resulted in reduced (*P*< 0.01) concentrations of follicular fluid triglycerides and plasma fatty acids (Fig. 7A and  B). Concentrations of l-carnitine in plasma and follicular fluid did not significantly differ with fluid type or diet supplementation (Fig. 7C).

### Experiment 3: effect of maternal diet on oocyte developmental potential

**Figure 8:**
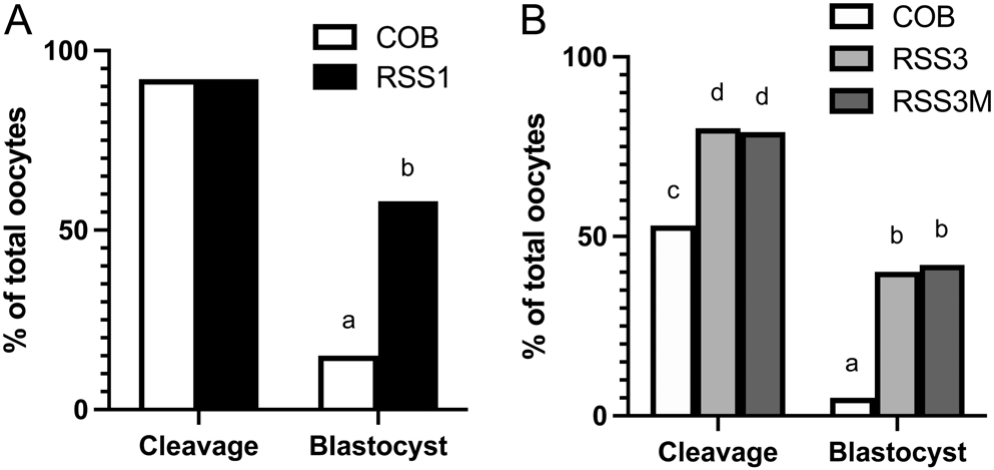
Embryonic development rates after intracytoplasmic sperm injection (ICSI). Cleavage rates (≥2 cell embryos per sperm-injected oocytes by 2 days after ICSI) and blastocyst rates (number of blastocysts per sperm-injected oocytes) for (A) oocytes from mares supplemented with grains (COB, * n* = 13) or a reproductive support supplement with additional antioxidants (RSS1, *n* = 12) and (B) oocytes from mares supplemented with grains (COB, *n* =19) or reproductive support supplements with n-3 PUFA (RSS3, *n* = 15) or with the substitution of n-3 PUFA with n-6 PUFA (RSS3M, *n* = 24). Bars with different superscripts differed (a,b; *P*< 0.05 or c,d; *P*< 0.1) for the same endpoint.

Oocyte developmental potential to the blastocyst stage was assessed between grain-fed control mares and mares fed RSS with additional antioxidants or with variable levels of omega-3 vs omega-6 fatty acids (see RSS for Experiments 1 and 3, Table 1). Cleavage rates of sperm-injected oocytes at 1 or 2 days after ICSI were similar for mares provided a grain supplement (12/13, 92%) or the RSS with additional antioxidants (11/12, 92%) (Fig. 8A); however, more blastocysts developed per sperm-injected oocytes by day 7 or 8 after ICSI for mares supplemented with the RSS than with grains (7/12, 58% and 2/13, 15%, respectively, *P*= 0.04) (Fig. 8A). When fatty acid concentrations were varied, cleavage rates were not significantly different among groups (Fig. 8B). The number of blastocysts per injected oocyte was similar for oocytes from mares fed the RSSs, regardless of omega-3 and omega-6 fatty acid content, but higher (*P*≤ 0.02) than for mares supplemented with grain (supplement with omega-3 fatty acids, 6/15, 40%; supplement with substitution of n-3 with n-6 fatty acids, 10/24, 42%; and grain supplementation, 1/19, 5%) (Fig. 8B). The results demonstrate that dietary components significantly affected the potential of oocytes to reach the blastocyst stage of development.

## Discussion

The extent that diet supplements can affect female fertility is dependent on their potential to influence reproductive tissues directly or indirectly, with the oocyte being one of the most important and difficult of cells to impact. In the present study, we examined the potential of dietary supplements, designed to support health and reproductive function and fed for approximately 2 months, to affect oocyte metabolic function, lipid content, and developmental potential in older mares. We used the mare, a monogastric large animal with an easily manipulated reproductive tract, for our studies. Maternal aging has a marked effect on mare reproductive efficiency, with a decline beginning in the early teen years (Ginther 1992). Although many mares will cycle into their 20s, their fertility is poor and associated with a decline in oocyte developmental competence (Carnevale & Ginther 1995). Considering the similarities between the mare and woman in follicular development (e.g. monovular, long follicular phase, similar follicle wave patterns, decades-long reproductive lifespans) and age-associated changes in reproduction, the mare represents an applicable model for reproductive aging in women (Carnevale 2008, Carnevale *et al.* 2020) and vice versa.

ARTs are often used in mares and women to produce offspring from subfertile females. However, in both species, oocyte developmental potential can be low, especially in older oocyte donors (Navot *et al.* 1991, Carnevale & Ginther 1995), and assisted reproductive procedures are costly and not always practicable. Diet supplementation represents a feasible approach *in vivo* to improve female reproductive outcomes, especially if developmental ability of the oocyte that is destined to ovulate can be improved. Specific compounds have been studied for their effect on reproductive parameters, although more information is available for women than mares. However, our primary goal was to determine if and to what extent the follicle and oocyte could be impacted by dietary supplements and not to study individual components, as nutrient function can be codependent and synergistic. The base diet for the studies was hay of a quality consistent with that fed to mares under maintenance conditions and for reproductively active mares. Treatment groups were provided a nutritional foundation of vitamins, minerals, pre-, and probiotics for digestive support, and proprietary RSSs with additional antioxidant and cell metabolic support nutrients. Some of these nutrients have been reported to improve stallion semen quality, including d-alpha-tocopheryl acetate, n-3 PUFAs, and l-carnitine (Deichsel *et al.* 2008, Contri *et al.* 2011, Schmid-Lausigk & Aurich 2014, Ruiz *et al.* 2021). The effects of these compounds on gamete quality in the mare have not been definitively assessed. Our study used multiple and novel endpoints to demonstrate that the dietary intervention resulted in both systemic and follicular effects, culminating in oocytes from older mares having significant changes in metabolic function, lipid composition, and developmental potential.

The follicle and associated cells support the oocyte and can provide an indication of oocyte quality. Elevated follicular concentrations of ROS can reflect the active metabolism of a healthy follicle (Zarezadeh *et al.* 2019); however, excessive levels of ROS are unfavorable for oocyte quality and embryo development, as observed for obesity, diabetes, and aging in women (Jančar *et al.* 2007, Karuputhula *et al.* 2013, Gu *et al.* 2015, Lai *et al.* 2018). The primary difference that we observed when older mares were fed a RSS with additional antioxidants instead of grain was a significant decrease in ROS production relative to aerobic metabolism in granulosa cells. Reduced ROS production by granulosa cells could indicate a healthier follicular environment for oocyte development and maturation, as oxidative stress has a major negative impact on female fertility and oocyte health (Devine *et al.* 2012). In this study, we only examined aerobic metabolism, although the extent that aerobic vs anaerobic metabolism is used by equine granulosa cells is not known. In the pig, another species having oocytes with abundant lipids, granulosa cells generate energy mostly via anaerobic glycolysis (Kansaku *et al.* 2017). Further studies are needed to determine the metabolic preferences of equine granulosa cells.

We used novel microsensors to examine aerobic and anaerobic metabolism in single oocyte and early embryos (Obeidat *et al.* 2018, 2019). Oocytes primarily generate energy through mitochondrial aerobic metabolism, resulting in the consumption of oxygen (May-Panloup *et al.* 2007). Aerobic metabolism, under physiological conditions, is controlled by energy demand and can be measured as basal OCR. The maximal aerobic metabolic potential provides information as to how much aerobic energy can be produced; it can be measured after the addition of mitochondrial uncoupler agents (Brand & Nicholls 2011). In the present study, oocyte basal aerobic metabolism was significantly higher for oocytes from mares fed the RSS than those fed grain, with aerobic metabolism of oocytes from supplemented mares comparable to the same endpoint in oocytes from young mares in a previous study (Catandi *et al.* 2021). In agreement with the previous study ( Catandi *et al.*2021), higher oocyte aerobic metabolic activity was associated with more oocytes capable of developing into blastocysts. Although basal aerobic metabolism was reduced in grain-fed mares, maximal aerobic metabolism was similar between the two groups. Consequently, oocytes from mares fed grain had a similar potential to produce energy through aerobic metabolism; however, they were using significantly less of their energy potential under basal conditions than oocytes from mares fed the RSS. In intact cells, basal mitochondrial metabolism is limited by substrate availability and regulated by energy demand (Brand & Nicholls 2011). Reduced basal oxygen consumption in oocytes from mares fed grain supplements may, therefore, be associated with altered energy sensing cellular mechanisms, such as AMP-activated protein kinase (AMPK). This enzyme senses energy status and regulates anabolic and catabolic pathways to equilibrate ATP production and substrate consumption inside the cell and to regulate progression of oocyte maturation (Abdulhasan *et al.* 2017, Yang *et al.* 2020). In oocytes, AMPK activity is altered by maternal metabolic dysfunctions, such as diabetes (Ratchford *et al.* 2007), and can be influenced by diet, although precise mechanisms have not been elucidated (Gu *et al.* 2015). Oocyte AMPK activity was not assayed in this study, but we speculate that diet supplementation with grains could have a negative impact, as seen with high-fat diets in multiple other tissues (Lindholm *et al.* 2013). In addition, ingredients in the RSS could have had a positive impact on AMPK function in oocytes, as seen after stimulation of lipid metabolism or addition of l-carnitine to oocyte *in vitro* maturation (Downs *et al.* 2009, Downs 2015). Higher oocyte basal mitochondrial metabolism and reduced lipid abundance observed after diet supplementation with the RSS are consistent with this postulation.

Basal anaerobic metabolism (based on ECAR) was similar for the two groups, indicating that the grain-fed mares’ oocytes did not try to compensate for lower aerobic metabolism by increasing anaerobic energy production. It remains unclear whether oocytes have the ability to recognize limitations in mitochondrial energy production and compensate by increasing their reliance on anaerobic glycolysis, as observed in muscle cells during intense exercise and hypoxia (Bowtell *et al.* 2014). Regardless, almost 90% of the energy produced in equine oocytes is provided by aerobic pathways (Lewis *et al.* 2020), and thus, anaerobic energy production would likely not be enough to compensate for mitochondrial dysfunction. Ultimately, this suggests that the oocytes from the mares that were provided the RSS had overall higher energy production than those fed grains. In addition, oocytes from mares fed the diet supplement were capable of more stimulated anaerobic metabolism, suggesting better metabolic flexibility to produce energy. Overall, oocytes from the grain-fed mares performed more anaerobic glycolysis as a proportion of their total energy production when compared to oocytes from mares fed the support supplement. This metabolic adaptation could affect embryo development, as the oocytes are using more of their glucose and pyruvate reserves when they should be more dependent on β-oxidation for energy production (Lewis *et al.* 2020).

Copy numbers of mtDNA were determined as an estimate of oocyte mitochondrial numbers, although the association between mtDNA copy numbers and oocyte metabolic potential is not well known. In the present study, mtDNA copy numbers were significantly higher in oocytes from mares fed grain than the RSS, although basal aerobic metabolism was higher in the latter. Oocytes are not able to activate mitophagy in response to mitochondrial damage (Boudoures *et al.* 2017); therefore, mitochondrial dysfunction and metabolic stress can lead to an abnormal, compensatory increase in mtDNA copy numbers (DiMauro & Schon 2003, Meldrum *et al.* 2016). In a previous study from our group, oocytes obtained from young vs old mares had higher basal and maximal aerobic metabolism, although no difference was noted for mtDNA copy numbers, confirming that oocyte mtDNA content is not indicative of mitochondrial function or oocyte quality (Catandi *et al.* 2021). Our results are consistent with findings in women. Mature oocytes from young women have fewer mtDNA copy numbers but greater mitochondrial membrane potential when compared to oocytes from older women (Pasquariello *et al.* 2019), further supporting that mtDNA copy numbers and mitochondria activity are not positively related.

Fatty acids have been suggested to be the primary substrate for oxidative energy production in equine oocytes (Lewis *et al.* 2020). The RSS provided additional PUFAs and l-carnitine. Within mitochondria, l-carnitine is essential for fatty acid β-oxidation, acting as a co-factor in the rate-limiting step involving the transport of activated fatty acids into mitochondria (Dunning & Robker 2012). Additional dietary lipids may serve as an energy reserve for the oocyte; however, supplementation of these fatty acids alone has been associated with more negative than positive effects on oocyte quality in other species (Zarezadeh *et al.* 2019). Short-term l-carnitine supplementation to ewes does not affect follicular development and ovulation, while l-carnitine combined with long-chain fatty acid supplementation improves the number and size of preovulatory follicles and ovulation rates (El-Shahat & Abo-El maaty 2010). Thus, there seems to be a synergistic effect of l-carnitine and fatty acids on the ovine ovaries and developing follicles. As lipid content varies in oocytes from different species (Dunning *et al.* 2014), the extent that fatty acids are used as an energy substrate for the oocyte and the impact of l-carnitine could vary.

In the first experiment, the RSS had additional antioxidants to offset the potential effects of aging, including CoQ10, pterostilbene, and PQQ. Aging impairs the expression of enzymes involved in the natural production of CoQ10 in multiple tissues, including follicular cells (Ben-Meir *et al.* 2015), and dietary supplementation with CoQ10 is associated with improved oocyte mitochondrial function and developmental potential for aged mice (Ben-Meir *et al.* 2015). Pterostilbene is associated with lowering the effects of oxidative stress in aging, and murine oocyte quality and maturation rates improve when supplemented during *in vitro* maturation (Li *et al.* 2018, Ullah *et al.* 2018). PQQ is a natural antioxidant that has been associated with improved reproductive performance when supplemented to female mice (Steinberg *et al.* 2003). In our study, a positive synergistic effect of the antioxidants with other components of the RSS could have occurred. However, results from all experiments strongly support that the overall diet supplement was the primary factor affecting oocytes, regardless of the addition of antioxidants.

In the second experiment, lipid content was compared for oocytes from the same mares prior to and after approximately 8 weeks of feeding RSS mixed with a pelleted complete feed and grain mix (RSS2). Oocyte lipid composition was altered after supplementation, with a pronounced reduction in triglyceride abundance. Lipids may serve as the main substrate for aerobic energy production during oocyte maturation, as glucose is mostly directed for anaerobic energy production (Lewis *et al.* 2020). Systemic and follicular triglyceride concentrations were consistent with a previous study from our group using the same methodology and follicle category (Sessions-Bresnahan *et al.* 2016), with TG concentrations higher in plasma when compared to follicular fluid, although this relationship was not consistent with other equine studies (Collins *et al.* 1997, Satué *et al.* 2019). Triglyceride concentrations in follicular fluid were reduced after diet supplementation, although a similar decline was not noted in systemic concentrations. However, the diet supplement caused a significant decline in systemic free fatty acids. Omega-3 PUFA supplementation has been associated with systemic hypolipidemic effects (Madsen *et al.* 1999), but it did not affect fatty acid concentrations in the serum of pregnant and lactating mares (Hodge *et al.* 2017). The concentrations of l-carnitine in the present study were consistent with previous reports in equine follicular fluid and plasma (Foster *et al.* 1988, Zeyner & Harmeyer 1999, Fernández-Hernández *et al.* 2020). A short-term increase in systemic l-carnitine occurs after oral ingestion in horses; however, plasma concentrations are only increased for a few hours after ingestion (Zeyner & Harmeyer 1999). We collected blood samples in the morning prior to the consumption of supplements, potentially missing any transitory increase in systemic l-carnitine; however, follicular fluid samples were collected in the late morning or early afternoon after supplements were fed in the morning. Therefore, although the results demonstrate that dietary supplementation altered oocyte lipid content, further studies are needed to determine if the effect was primarily caused by follicular or systemic alterations.

Basal and maximal OCR from early embryos were consistently higher than values observed for oocytes, as noted in a previous study (Catandi *et al.* 2021). During early embryonic development, mitochondrial numbers do not change (Hendriks *et al.* 2019), but the organelles go through morphological changes from the immature stage present in oocytes to more active stages (Bavister & Squirrell 2000, Van Blerkom 2011). However, no differences were observed between diet groups for day-2 embryo aerobic metabolism.

In the present experiments, oocyte developmental potential was determined by cleavage and blastocyst formation after ICSI. In our first experiment, cleavage and blastocyst rates were compared for mares that were fed grain or the diet supplement with additional antioxidants. Cleavage rates were not significantly different between groups, consistent with our previous finding when comparing cleavage rates after ICSI for young and old mares (Catandi *et al.* 2021). However, blastocyst formation was significantly improved for mares fed RSS when compared to grain. Considering the mean age of the mares (18.5 years), the blastocyst rate for mares fed the RSS (58%) was high when compared to rates obtained in a previous study using frozen-thawed sperm from the same stallion for ICSI (21% for old mares, ≥20 years and 48% for young mares, ≤14 years) (Catandi *et al.* 2021). In the final experiment, the developmental potential of oocytes from mares fed grain was compared to mares fed the RSS or the same supplement after the substitution of most of the n-3 PUFAs with n-6 PUFAs. Regardless of the PUFA content, mares provided the diet supplement had similar cleavage and blastocyst formation rates. In agreement with these findings, embryonic development rates after *in vitro* fertilization of oocytes from dairy cows supplemented with n-3 PUFA for 3 months were higher when compared to cows fed a control diet but not different from cows supplemented with n-6 PUFA (Zachut *et al.* 2010). However, mares fed grain tended to have lower cleavage rate and had a significantly lower blastocyst formation rate, demonstrating that the final concentration of n-3 or n-6 PUFA is not as crucial as other ingredients in the dietary supplementation for improving the developmental potential of oocytes from older mares.

In the current study, we did not try to identify the effect of one single nutrient on mare follicular metabolism and oocyte developmental potential. Instead, we compared supplementation with grain products, which remain popular feed ingredients in the equine industry and the Western human diet, to feed ingredients designed to support overall wellness and potentially support reproduction and mitochondrial function. Because we used a complex of nutrients, we cannot differentiate individual vs synergistic effects of supplement components on the associated differences in oocyte metabolic function and lipid composition. We are also unsure of the extent that the complex nutrients were beneficial as opposed to even a limited amount of grain was detrimental to oocyte metabolism and developmental potential. However, we clearly observed that the diet components had a substantive effect on oocyte composition, metabolic function, and developmental potential. Consequently, we demonstrated that short-term diet additives can affect reproductive function at the cellular level in older mares, providing a feasible method and model to study the interaction of diet and reproduction in the female. Our results suggest that diet has the potential to alter reproductive outcomes in mares by ultimately having a direct effect on the ovarian follicle and oocyte.

## Supplementary Material

Supplementary Table 1. Abundance of oocyte lipids that were affected by mare diet supplementation with RSS2. Single oocytes were analyzed from mares Pre and Post approximately two months of supplementation. Results are presented as mean ± SEM.

Supplementary Table 2. Abundance of oocyte metabolites that were affected by supplementation with RSS2. Single oocytes were analyzed from the same mares Pre and Post approximately two months of supplementation. Results are presented as mean ± SEM.

Supplementary Figure 1. Aerobic metabolism, based on oxygen consumption rates (OCR), in embryos 2 days after intracytoplasmic sperm injection of oocytes from older mares supplemented with grain and corn oil (COB) or complex nutrients to support health and reproductive function (RSS1). (A) Basal OCR (COB, n=11; RSS1, n=12) and (B) maximal OCR (COB, n=6; RSS1, n=6). Barcharts present means ± SEMs.

## Declaration of interest

The authors declare that there is no conflict of interest that could be perceived as prejudicing the impartiality of the research reported.

## Funding

This work was collectively supported by the Cecil and Irene Hylton Foundation http://dx.doi.org/10.13039/100016177, OEDIT Advanced Industries Accelerator POC Program grant, the National Institute of Health (grant number 1R21HD097601-01), National Science Foundation http://dx.doi.org/10.13039/100000001 Grants No. 0841259 and 1450032, and Animal Health and Disease Grant No. COLV2019-5/Project Accession No. 1020546 from the USDA National Institute of Food and Agriculture http://dx.doi.org/10.13039/100005825.

## Author contribution statement

E C initiated experimental designs, general organization, and base funding. A C and T C participated in study design, additional funding, and supervision of microsensor and O2K assays. G C and E C performed mare procedures. L M participated in sample collections and experiment organization for Experiments 1 and 2. T C and Y O developed and provided microsensors, and G C and Y O performed microsensor assays. L L and A C performed O2K assays. C B conducted lipidomic assays. G C, C B and L L analyzed data. G C and E C prepared the manuscript, which was edited by all authors.

## References

[bib1] Aardema H Lolicato F van de Lest CHA Brouwers JF Vaandrager AB van Tol HTA Roelen BAJ Vos PLAM Helms JB Gadella BM 2013 Bovine cumulus cells protect maturing oocytes from increased fatty acid levels by massive intracellular lipid storage. Biology of Reproduction 88 164–164. (10.1095/biolreprod.112.106062) 23616596

[bib2] Abdulhasan MK Li Q Dai J Abu-Soud HM Puscheck EE Rappolee DA 2017 CoQ10 increases mitochondrial mass and polarization, ATP and Oct4 potency levels, and bovine oocyte MII during IVM while decreasing AMPK activity and oocyte death. Journal of Assisted Reproduction and Genetics 34 1595–1607. (10.1007/s10815-017-1027-y) 28900834 PMC5714820

[bib3] Babayev E Seli E 2015 Oocyte mitochondrial function and reproduction. Current Opinion in Obstetrics and Gynecology 27 175–181. (10.1097/GCO.0000000000000164) 25719756 PMC4590773

[bib4] Bavister BD Squirrell JM 2000 Mitochondrial distribution and function in oocytes and early embryos. Human Reproduction 15 (Supplement 2) 189–198. (10.1093/humrep/15.suppl_2.189) 11041524

[bib5] Ben-Meir A Burstein E Borrego-Alvarez A Chong J Wong E Yavorska T Naranian T Chi M Wang Y Bentov Y 2015 Coenzyme Q10 restores oocyte mitochondrial function and fertility during reproductive aging. Aging Cell 14 887–895. (10.1111/acel.12368) 26111777 PMC4568976

[bib6] Boudoures AL Saben J Drury A Scheaffer S Modi Z Zhang W Moley KH 2017 Obesity-exposed oocytes accumulate and transmit damaged mitochondria due to an inability to activate mitophagy. Developmental Biology 426 126–138. (10.1016/j.ydbio.2017.04.005) 28438607

[bib7] Bowtell JL Cooke K Turner R Mileva KN Sumners DP 2014 Acute physiological and performance responses to repeated sprints in varying degrees of hypoxia. Journal of Science and Medicine in Sport 17 399–403. (10.1016/j.jsams.2013.05.016) 23809839

[bib8] Brand MD Nicholls DG 2011 Assessing mitochondrial dysfunction in cells. Biochemical Journal 435 297–312. (10.1042/BJ20110162) 21726199 PMC3076726

[bib9] Broughton DE Moley KH 2017 Obesity and female infertility: potential mediators of obesity’s impact. Fertility and Sterility 107 840–847. (10.1016/j.fertnstert.2017.01.017) 28292619

[bib10] Carnevale EM 2008 The mare model for follicular maturation and reproductive aging in the woman. Theriogenology 69 23–30. (10.1016/j.theriogenology.2007.09.011) 17976712

[bib11] Carnevale EM 2016 Advances in collection, transport and maturation of equine oocytes for assisted reproductive techniques. Veterinary Clinics of North America: Equine Practice 32 379–399. (10.1016/j.cveq.2016.07.002) 27726987

[bib12] Carnevale EM Ginther OJ 1995 Defective oocytes as a cause of subfertility in old mares. Biology of Reproduction 52 209–214. (10.1093/biolreprod/52.monograph_series1.209)

[bib13] Carnevale EM Metcalf ES 2019 Morphology, developmental stages and quality parameters of in vitro-produced equine embryos. Reproduction, Fertility, and Development 31 1758–1770. (10.1071/RD19257) 31718765

[bib14] Carnevale EM Catandi GD Fresa K 2020 Equine aging and the oocyte: a potential model for reproductive aging in women. Journal of Equine Veterinary Science 89 103022. (10.1016/j.jevs.2020.103022) 32563447

[bib15] Catandi G Obeidat Y Chicco A Chen T Carnevale E 2019 167 Basal and maximal oxygen consumption of oocytes from young and old mares. Reproduction, Fertility and Development 31 208–208. (10.1071/RDv31n1Ab167)

[bib16] Catandi GD Obeidat YM Broeckling CD Chen TW Chicco AJ Carnevale EM 2021 Equine maternal aging affects oocyte lipid content, metabolic function and developmental potential. Reproduction 161 399–409. (10.1530/REP-20-0494) 33539317 PMC7969451

[bib17] Cecchino GN Seli E Alves da Motta EL García-Velasco JA 2018 The role of mitochondrial activity in female fertility and assisted reproductive technologies: overview and current insights. Reproductive Biomedicine Online 36 686–697. (10.1016/j.rbmo.2018.02.007) 29598846

[bib18] Chicco AJ Le CH Gnaiger E Dreyer HC Muyskens JB D’Alessandro A Nemkov T Hocker AD Prenni JE Wolfe LM 2018 Adaptive remodeling of skeletal muscle energy metabolism in high-altitude hypoxia: lessons from AltitudeOmics. Journal of Biological Chemistry 293 6659–6671. (10.1074/jbc.RA117.000470) 29540485 PMC5936810

[bib19] Collins A Palmer E Bézard J Burke J Duchamp G Buckley T 1997 A comparison of the biochemical composition of equine follicular fluid and serum at four different stages of the follicular cycle. Equine Veterinary Journal: Supplement 29 12–16. (10.1111/j.2042-3306.1997.tb05092.x) 9593520

[bib20] Contri A De Amicis I Molinari A Faustini M Gramenzi A Robbe D Carluccio A 2011 Effect of dietary antioxidant supplementation on fresh semen quality in stallion. Theriogenology 75 1319–1326. (10.1016/j.theriogenology.2010.12.003) 21295825

[bib21] Dalbies-Tran R Cadoret V Desmarchais A Elis S Maillard V Monget P Monniaux D Reynaud K Saint-Dizier M Uzbekova S 2020 A comparative analysis of oocyte development in mammals. Cells 9 1002. (10.3390/cells9041002) PMC7226043 32316494

[bib22] Das UN 2006 Essential fatty acids: biochemistry, physiology and pathology. Biotechnology Journal 1 420–439. (10.1002/biot.200600012) 16892270

[bib23] de Lorgeril M Salen P 2012 New insights into the health effects of dietary saturated and omega-6 and omega-3 polyunsaturated fatty acids. BMC Medicine 10 50. (10.1186/1741-7015-10-50) PMC3394202 22613931

[bib24] Deichsel K Palm F Koblischke P Budik S Aurich C 2008 Effect of a dietary antioxidant supplementation on semen quality in pony stallions. Theriogenology 69 940–945. (10.1016/j.theriogenology.2008.01.007) 18358523

[bib25] Devine PJ Perreault SD Luderer U 2012 Roles of reactive oxygen species and antioxidants in ovarian toxicity. Biology of Reproduction 86 27. (10.1095/biolreprod.111.095224) PMC3290661 22034525

[bib26] Dhungana S Carlson JE Pathmasiri W McRitchie S Davis M Sumner S Appt SE 2016 Impact of a western diet on the ovarian and serum metabolome. Maturitas 92 134–142. (10.1016/j.maturitas.2016.07.008) 27621251

[bib27] DiMauro S Schon EA 2003 Mitochondrial respiratory-chain diseases. New England Journal of Medicine 348 2656–2668. (10.1056/NEJMra022567) 12826641

[bib28] Downs SM 2015 Nutrient pathways regulating the nuclear maturation of mammalian oocytes. Reproduction, Fertility, and Development 27 572–582. (10.1071/RD14343) 25798589

[bib29] Downs SM Mosey JL Klinger J 2009 Fatty acid oxidation and meiotic resumption in mouse oocytes. Molecular Reproduction and Development 76 844–853. (10.1002/mrd.21047) 19455666 PMC3995453

[bib30] Dunning KR Robker RL 2012 Promoting lipid utilization with l-carnitine to improve oocyte quality. Animal Reproduction Science 134 69–75. (10.1016/j.anireprosci.2012.08.013) 22917873

[bib31] Dunning KR Russell DL Robker RL 2014 Lipids and oocyte developmental competence: the role of fatty acids and β-oxidation. Reproduction 148 R15–R27. (10.1530/REP-13-0251) 24760880

[bib32] El-Shahat KH Abo-El maaty AM 2010 The effect of dietary supplementation with calcium salts of long chain fatty acids and/or l-carnitine on ovarian activity of Rahmani ewes. Animal Reproduction Science 117 78–82. (10.1016/j.anireprosci.2009.04.005) 19473790

[bib33] Fernández-Hernández P Sánchez-Calabuig MJ García-Marín LJ Bragado MJ Gutiérrez-Adán A Millet Ó Bruzzone C González-Fernández L Macías-García B 2020 Study of the metabolomics of equine preovulatory follicular fluid: a way to improve current in vitro maturation media. Animals 10 883. (10.3390/ani10050883) PMC7278476 32438699

[bib34] Foster CVL Harris RC Snow DH 1988 The effect of oral l-carnitine supplementation on the muscle and plasma concentrations in the thoroughbred horse. Comparative Biochemistry and Physiology: A, Comparative Physiology 91 827–835. (10.1016/0300-9629(8890971-1) 2907450

[bib35] Frape DL 2004 Equine Nutrition and Feeding. Oxford, UK; Ames, IA: Blackwell Publishing.

[bib36] Gaskins AJ Chavarro JE 2018 Diet and fertility: a review. American Journal of Obstetrics and Gynecology 218 379–389. (10.1016/j.ajog.2017.08.010) 28844822 PMC5826784

[bib37] Ginther O 1992 Reproductive biology of the mare. In Basic and Applied Aspects, vol. 75. Cross Plains, Wis.: Equiservices.

[bib38] Gonzalez-Castro RA Carnevale EM 2018 Association of equine sperm population parameters with outcome of intracytoplasmic sperm injections. Theriogenology 119 114–120. (10.1016/j.theriogenology.2018.06.027) 30006126

[bib39] Goo S Pham T Han JC Nielsen P Taberner A Hickey A Loiselle D 2013 Multiscale measurement of cardiac energetics. Clinical and Experimental Pharmacology and Physiology 40 671–681. (10.1111/1440-1681.12139) 23745944

[bib40] Gu L Liu H Gu X Boots C Moley KH Wang Q 2015 Metabolic control of oocyte development: linking maternal nutrition and reproductive outcomes. Cellular and Molecular Life Sciences 72 251–271. (10.1007/s00018-014-1739-4) 25280482 PMC4389777

[bib41] Hallebeek JM Beynen AC 2002 Dietary fats and lipid metabolism in relation to equine health, performance and disease. *PhD Thesis*. The Netherlands: Department of Nutrition, Utrecht University.

[bib42] Hashimoto S Morimoto N Yamanaka M Matsumoto H Yamochi T Goto H Inoue M Nakaoka Y Shibahara H Morimoto Y 2017 Quantitative and qualitative changes of mitochondria in human preimplantation embryos. Journal of Assisted Reproduction and Genetics 34 573–580. (10.1007/s10815-017-0886-6) 28190213 PMC5427646

[bib43] Hendriks WK Colleoni S Galli C Paris DBBP Colenbrander B Stout TAE 2019 Mitochondrial DNA replication is initiated at blastocyst formation in equine embryos. Reproduction, Fertility, and Development 31 570–578. (10.1071/RD17387) 30423285

[bib44] Hess T Ross-Jones T 2014 Omega-3 fatty acid supplementation in horses. Revista Brasileira de Zootecnia 43 677–683. (10.1590/S1516-35982014001200008)

[bib45] Hodge LB Rude BJ Dinh TN Lemley CO 2017 Effect of ω-3 fatty acid supplementation to gestating and lactating mares: on milk IgG, mare and foal blood concentrations of IgG, insulin and glucose, placental efficiency, and fatty acid composition of milk and serum from mares and foals. Journal of Equine Veterinary Science 51 70–78. (10.1016/j.jevs.2016.11.014)

[bib46] Jahangirifar M Taebi M Nasr-Esfahani MH Askari GH 2019 Dietary patterns and the outcomes of assisted reproductive techniques in women with primary infertility: a prospective cohort study. International Journal of Fertility and Sterility 12 316–323. (10.22074/ijfs.2019.5373) 30291693 PMC6186288

[bib47] Jančar N Kopitar AN Ihan A Klun IV Bokal EV 2007 Effect of apoptosis and reactive oxygen species production in human granulosa cells on oocyte fertilization and blastocyst development. Journal of Assisted Reproduction and Genetics 24 91–97. (10.1007/s10815-006-9103-8) 17216562 PMC3454987

[bib48] Kansaku K Itami N Kawahara-Miki R Shirasuna K Kuwayama T Iwata H 2017 Differential effects of mitochondrial inhibitors on porcine granulosa cells and oocytes. Theriogenology 103 98–103. (10.1016/j.theriogenology.2017.07.049) 28779614

[bib49] Karuputhula NB Chattopadhyay R Chakravarty B Chaudhury K 2013 Oxidative status in granulosa cells of infertile women undergoing IVF. Systems Biology in Reproductive Medicine 59 91–98. (10.3109/19396368.2012.743197) 23278116

[bib50] Kermack AJ Lowen P Wellstead SJ Fisk HL Montag M Cheong Y Osmond C Houghton FD Calder PC Macklon NS 2020 Effect of a 6-week ‘Mediterranean’ dietary intervention on in vitro human embryo development: the preconception dietary supplements in assisted reproduction double-blinded randomized controlled trial. Fertility and Sterility 113 260–269. (10.1016/j.fertnstert.2019.09.041) 31870562

[bib51] Lai Q Xiang W Li Q Zhang H Li Y Zhu G Xiong C Jin L 2018 Oxidative stress in granulosa cells contributes to poor oocyte quality and IVF-ET outcomes in women with polycystic ovary syndrome. Frontiers of Medicine 12 518–524. (10.1007/s11684-017-0575-y) 29260383

[bib52] Lewis N Hinrichs K Leese HJ McG Argo C Brison DR Sturmey R 2020 Energy metabolism of the equine cumulus oocyte complex during in vitro maturation. Scientific Reports 10 3493. (10.1038/s41598-020-60624-z) PMC7044441 32103136

[bib53] Li YR Li S Lin CC 2018 Effect of resveratrol and pterostilbene on aging and longevity: Effect of resveratrol and pterostilbene on aging and longevity. BioFactors 44 69–82. (10.1002/biof.1400) 29210129

[bib54] Lindholm CR Ertel RL Bauwens JD Schmuck EG Mulligan JD Saupe KW 2013 A high-fat diet decreases AMPK activity in multiple tissues in the absence of hyperglycemia or systemic inflammation in rats. Journal of Physiology and Biochemistry 69 165–175. (10.1007/s13105-012-0199-2) 22941749 PMC3644018

[bib55] Lolicato F Brouwers JF de Lest van CHA Wubbolts R Aardema H Priore P Roelen BAJ Helms JB Gadella BM 2015 The cumulus cell layer protects the bovine maturing oocyte against fatty acid-induced lipotoxicity. Biology of Reproduction 92 16. (10.1095/biolreprod.114.120634) 25297544

[bib56] Madsen L Rustan AC Vaagenes H Berge K Dyrøy E Berge RK 1999 Eicosapentaenoic and docosahexaenoic acid affect mitochondrial and peroxisomal fatty acid oxidation in relation to substrate preference. Lipids 34 951–963. (10.1007/s11745-999-0445-x) 10574660

[bib57] May-Panloup P Chretien MF Malthiery Y Reynier P 2007 Mitochondrial DNA in the oocyte and the developing embryo. Current Topics in Developmental Biology 77 51–83. (10.1016/S0070-2153(0677003-X) 17222700

[bib58] Meldrum DR Casper RF Diez-Juan A Simon C Domar AD Frydman R 2016 Aging and the environment affect gamete and embryo potential: can we intervene? Fertility and Sterility 105 548–559. (10.1016/j.fertnstert.2016.01.013) 26812244

[bib59] Navot D Bergh PA Williams MA Garrisi GJ Guzman I Sandler B Grunfeld L 1991 Poor oocyte quality rather than implantation failure as a cause of age-related decline in female fertility. Lancet 337 1375–1377. (10.1016/0140-6736(9193060-m) 1674764

[bib60] Nehra D Le HD Fallon EM Carlson SJ Woods D White YA Pan AH Guo L Rodig SJ Tilly JL 2012 Prolonging the female reproductive lifespan and improving egg quality with dietary omega-3 fatty acids. Aging Cell 11 1046–1054. (10.1111/acel.12006) 22978268 PMC5624332

[bib61] Obeidat YM Evans AJ Tedjo W Chicco AJ Carnevale E Chen TW 2018 Monitoring oocyte/embryo respiration using electrochemical-based oxygen sensors. Sensors and Actuators: Part B 276 72–81. (10.1016/j.snb.2018.07.157)

[bib62] Obeidat YM Cheng MH Catandi G Carnevale E Chicco AJ Chen TW 2019 Design of a multi-sensor platform for integrating extracellular acidification rate with multi-metabolite flux measurement for small biological samples. Biosensors and Bioelectronics 133 39–47. (10.1016/j.bios.2019.02.069) 30909011 PMC6660976

[bib63] Pasquariello R Ermisch AF Silva E McCormick S Logsdon D Barfield JP Schoolcraft WB Krisher RL 2019 Alterations in oocyte mitochondrial number and function are related to spindle defects and occur with maternal aging in mice and humans. Biology of Reproduction 100 971–981. (10.1093/biolre/ioy248) 30476005

[bib64] Ratchford AM Chang AS Chi MM-Y Sheridan R Moley KH 2007 Maternal diabetes adversely affects AMP-activated protein kinase activity and cellular metabolism in murine oocytes. American Journal of Physiology: Endocrinology and Metabolism 293 E1198–E1206. (10.1152/ajpendo.00097.2007) 17684106

[bib65] Richani D Dunning KR Thompson JG Gilchrist RB 2021 Metabolic co-dependence of the oocyte and cumulus cells: essential role in determining oocyte developmental competence. Human Reproduction Update 27 27–47. (10.1093/humupd/dmaa043) 33020823

[bib66] Ruiz AJ Tibary A Heaton RA Hargreaves IP Leadon DP Bayly WM 2021 Effects of feeding coenzyme Q10-Ubiquinol on plasma coenzyme Q10 concentrations and semen quality in stallions. Journal of Equine Veterinary Science 96 103303. (10.1016/j.jevs.2020.103303) 33349408

[bib67] Satué K Fazio E Ferlazzo A Medica P 2019 Hematochemical patterns in follicular fluid and blood stream in cycling mares: a comparative note. Journal of Equine Veterinary Science 80 20–26. (10.1016/j.jevs.2019.06.016) 31443828

[bib68] Schmid-Lausigk Y Aurich C 2014 Influences of a diet supplemented with linseed oil and antioxidants on quality of equine semen after cooling and cryopreservation during winter. Theriogenology 81 966–973. (10.1016/j.theriogenology.2014.01.021) 24576708

[bib69] Sessions-Bresnahan DR Schauer KL Heuberger AL Carnevale EM 2016 Effect of obesity on the preovulatory follicle and lipid fingerprint of equine oocytes. Biology of Reproduction 94 15. (10.1095/biolreprod.115.130187) 26632608

[bib70] Siu MKY Cheng CY 2013 The blood-follicle barrier (BFB) in disease and in ovarian function. In Biology and Regulation of Blood-Tissue Barriers, pp. 186–192. Ed Cheng CY New York, NY: Springer. (10.1007/978-1-4614-4711-5_9) PMC4169694 23397625

[bib71] Smith CA Want EJ O’Maille G Abagyan R Siuzdak G 2006 XCMS: processing mass spectrometry data for metabolite profiling using nonlinear peak alignment, matching, and identification. Analytical Chemistry 78 779–787. (10.1021/ac051437y) 16448051

[bib72] Spikings EC Alderson J St John JC 2007 Regulated mitochondrial DNA replication during oocyte maturation is essential for successful porcine embryonic development. Biology of Reproduction 76 327–335. (10.1095/biolreprod.106.054536) 17035641

[bib73] Steinberg F Stites TE Anderson P Storms D Chan I Eghbali S Rucker R 2003 Pyrroloquinoline quinone improves growth and reproductive performance in mice fed chemically defined diets. Experimental Biology and Medicine 228 160–166. (10.1177/153537020322800205) 12563022

[bib74] Tautenhahn R Böttcher C Neumann S 2008 Highly sensitive feature detection for high resolution LC/MS. BMC Bioinformatics 9 504. (10.1186/1471-2105-9-504) PMC2639432 19040729

[bib75] Ullah O Zhongshu L Ali I Xu L Liu H Fang N 2018 Effects of pterostilbene on the activation of nuclear factor erythroid 2-related factor 2 pathway during in vitro maturation of mouse oocytes. Journal of Agricultural Science 10 35. (10.5539/jas.v10n7p35)

[bib76] Van Blerkom J 2011 Mitochondrial function in the human oocyte and embryo and their role in developmental competence. Mitochondrion 11 797–813. (10.1016/j.mito.2010.09.012) 20933103

[bib77] Wai T Ao A Zhang X Cyr D Dufort D Shoubridge EA 2010 The role of mitochondrial DNA copy number in mammalian fertility. Biology of Reproduction 83 52–62. (10.1095/biolreprod.109.080887) 20130269 PMC2888963

[bib78] Williams CJ Erickson GF 2012 Morphology and Physiology of the Ovary. In Endotext. Editors: KR Feingold, B Anawalt, A Boyce et al. South Dartmouth, MA, USA: MDText.com, Inc; 2000 25905186

[bib79] Yang W Wang L Wang F Yuan S 2020 Roles of AMP-activated protein kinase (AMPK) in mammalian reproduction. Frontiers in Cell and Developmental Biology 8 593005. (10.3389/fcell.2020.593005) 33330475 PMC7710906

[bib80] Zachut M Dekel I Lehrer H Arieli A Arav A Livshitz L Yakoby S Moallem U 2010 Effects of dietary fats differing in n-6:n-3 ratio fed to high-yielding dairy cows on fatty acid composition of ovarian compartments, follicular status, and oocyte quality. Journal of Dairy Science 93 529–545. (10.3168/jds.2009-2167) 20105525

[bib81] Zarezadeh R Mehdizadeh A Leroy JLMR Nouri M Fayezi S Darabi M 2019 Action mechanisms of n-3 polyunsaturated fatty acids on the oocyte maturation and developmental competence: potential advantages and disadvantages. Journal of Cellular Physiology 234 1016–1029. (10.1002/jcp.27101) 30073662

[bib82] Zeyner A Harmeyer J 1999 Metabolic functions of L-carnitine and its effects as feed additive in horses. A review. Archiv Fur Tierernahrung 52 115–138. (10.1080/17450399909386157) 10548966

