# Supplementary material for: Oocyte metabolic function, lipid composition, and developmental potential are altered by diet in older mares
Source: Reproduction. 2022 Jan 28;163(4):183–98. doi: 10.1530/REP-21-0351 (PMC8942336; doi:10.1530/REP-21-0351)
Supplement: Supplementary Table 1. Abundance of oocyte lipids that were affected by mare diet supplementation with RSS2. Single oocytes were analyzed from mares Pre and Post approximately two months of supplementation. Results are presented as mean ± SEM. [file supplementary_table_1.pdf]

Supplementary Table 1. Abundance of oocyte lipids that were affected by mare diet supplementation with RSS2. Single oocytes were analyzed from mares Pre and Post approximately two months of supplementation. Results are presented as mean ± SEM.

Lipid class	Lipid species	Pre	Post	P value
Triacylglycerols	TG(52:2)	$5.40 \times 10^6 \pm 0.42$	$2.71 \times 10^6 \pm 0.63$	0.049
	TG(52:3)	$4.27 \times 10^6 \pm 0.32$	$2.07 \times 10^6 \pm 0.49$	0.041
	TG(52:4)	$4.06 \times 10^6 \pm 0.35$	$2.26 \times 10^6 \pm 0.46$	0.047
	TG(50:2)	$3.52 \times 10^6 \pm 0.32$	$1.66 \times 10^6 \pm 0.35$	0.042
	TG(50:1)	$2.85 \times 10^6 \pm 0.34$	$1.36 \times 10^6 \pm 0.23$	0.049
	TG(54:3)	$2.34 \times 10^6 \pm 0.26$	$1.11 \times 10^6 \pm 0.27$	0.041
	TG(52:3)	$2.20 \times 10^6 \pm 0.16$	$1.11 \times 10^6 \pm 0.20$	0.039
	TG(52:2)	$2.02 \times 10^6 \pm 0.16$	$0.92 \times 10^6 \pm 0.19$	0.039
	TG(52:1)	$1.71 \times 10^6 \pm 0.16$	$0.79 \times 10^6 \pm 0.18$	0.039
	TG(50:3)	$1.65 \times 10^6 \pm 0.14$	$0.86 \times 10^6 \pm 0.18$	0.049
	TG(54:2)	$1.34 \times 10^6 \pm 0.17$	$0.60 \times 10^6 \pm 0.15$	0.041
	TG(56:7)	$0.91 \times 10^6 \pm 0.12$	$0.38 \times 10^6 \pm 0.08$	0.041
	TG(58:7)	$0.83 \times 10^6 \pm 0.09$	$0.41 \times 10^6 \pm 0.08$	0.039
	TG(50:4)	$0.81 \times 10^6 \pm 0.07$	$0.45 \times 10^6 \pm 0.10$	0.05
	TG(56:6)	$0.63 \times 10^6 \pm 0.09$	$0.24 \times 10^6 \pm 0.05$	0.039
	TG(54:7)	$0.60 \times 10^6 \pm 0.05$	$0.33 \times 10^6 \pm 0.07$	0.049
	TG(54:5)	$0.53 \times 10^6 \pm 0.04$	$0.26 \times 10^6 \pm 0.05$	0.039
	TG(51:2)	$0.47 \times 10^6 \pm 0.03$	$0.22 \times 10^6 \pm 0.04$	0.036
	TG(58:8)	$0.39 \times 10^6 \pm 0.06$	$0.16 \times 10^6 \pm 0.04$	0.047
	TG(49:2)	$0.37 \times 10^6 \pm 0.03$	$0.22 \times 10^6 \pm 0.04$	0.041
	TG(49:1)	$0.34 \times 10^6 \pm 0.02$	$0.18 \times 10^6 \pm 0.03$	0.044
	TG(51:3)	$0.33 \times 10^6 \pm 0.03$	$0.16 \times 10^6 \pm 0.03$	0.039
	TG(51:1)	$0.24 \times 10^6 \pm 0.02$	$0.12 \times 10^6 \pm 0.03$	0.041
	TG(53:2)	$0.23 \times 10^6 \pm 0.02$	$0.11 \times 10^6 \pm 0.02$	0.039
	TG(60:9)	$0.22 \times 10^6 \pm 0.02$	$0.11 \times 10^6 \pm 0.02$	0.036
	TG(52:2)	$0.21 \times 10^6 \pm 0.03$	$0.06 \times 10^6 \pm 0.02$	0.042
	TG(58:10)	$0.21 \times 10^6 \pm 0.02$	$0.11 \times 10^6 \pm 0.02$	0.041
	TG(52:2)	$0.18 \times 10^6 \pm 0.02$	$0.07 \times 10^6 \pm 0.02$	0.041
	TG(56:2)	$0.15 \times 10^6 \pm 0.01$	$0.07 \times 10^6 \pm 0.01$	0.048
	TG(51:5)	$0.14 \times 10^6 \pm 0.01$	$0.07 \times 10^6 \pm 0.01$	0.041
	TG(49:3)	$0.13 \times 10^6 \pm 0.01$	$0.07 \times 10^6 \pm 0.01$	0.048
	TG(60:2)	$0.12 \times 10^6 \pm 0.01$	$0.06 \times 10^6 \pm 0.02$	0.05
	TG(55:7)	$0.11 \times 10^6 \pm 0.01$	$0.06 \times 10^6 \pm 0.01$	0.041
	TG(52:1)	$0.10 \times 10^6 \pm 0.01$	$0.03 \times 10^6 \pm 0.008$	0.041
	TG(48:1)	$0.05 \times 10^6 \pm 0.01$	$0.02 \times 10^6 \pm 0.004$	0.05
	TG(60:6)	$0.02 \times 10^6 \pm 0.003$	$0.009 \times 10^6 \pm 0.002$	0.041
	TG(73:0)	$0.016 \times 10^6 \pm 0.001$	$0.009 \times 10^6 \pm 0.001$	0.041
	TG(56:3)	$0.013 \times 10^6 \pm 0.001$	$0.007 \times 10^6 \pm 0.0008$	0.039
	TG(53:1)	$0.007 \times 10^6 \pm 0.001$	$0.003 \times 10^6 \pm 0.0006$	0.048
Diacylglycerols	DG(36:2)	$3.55 \times 10^5 \pm 0.46$	$1.34 \times 10^5 \pm 0.30$	0.039
	DG(32:1)	$2.67 \times 10^5 \pm 0.39$	$1.17 \times 10^5 \pm 0.35$	0.039
	DG(36:2)	$2.23 \times 10^5 \pm 0.29$	$0.80 \times 10^5 \pm 0.15$	0.039
	DG(44:9)	$1.95 \times 10^5 \pm 0.26$	$0.71 \times 10^5 \pm 0.10$	0.039
	DG(40:8)	$1.74 \times 10^5 \pm 0.36$	$0.46 \times 10^5 \pm 0.11$	0.039
	DG(36:4)	$1.51 \times 10^5 \pm 0.23$	$0.64 \times 10^5 \pm 0.15$	0.05
	DG(34:2)	$1.32 \times 10^5 \pm 0.17$	$0.49 \times 10^5 \pm 0.10$	0.036

	DG(36:2)	$1.07 \times 10^5 \pm 0.08$	$0.57 \times 10^5 \pm 0.10$	0.039
	DG(40:8)	$0.84 \times 10^5 \pm 0.12$	$0.27 \times 10^5 \pm 0.04$	0.039
	DG(40:7)	$0.80 \times 10^5 \pm 0.17$	$0.38 \times 10^5 \pm 0.07$	0.05
	DG(38:4)	$0.74 \times 10^5 \pm 0.14$	$0.35 \times 10^5 \pm 0.08$	0.039
	DG(40:0)	$0.73 \times 10^5 \pm 0.08$	$0.35 \times 10^5 \pm 0.06$	0.039
	DG(40:9)	$0.64 \times 10^5 \pm 0.10$	$0.31 \times 10^5 \pm 0.06$	0.039
Monoacylglycerols	MG(16:0)	$3.35 \times 10^5 \pm 0.28$	$1.94 \times 10^5 \pm 0.24$	0.044
	MG(18:0)	$0.48 \times 10^5 \pm 0.04$	$0.29 \times 10^5 \pm 0.03$	0.048
Fatty acyls	6-cis-docosenamide	$0.48 \times 10^5 \pm 0.04$	$0.29 \times 10^5 \pm 0.03$	0.048
	12,15-cis-squamostatin A	$1.00 \times 10^6 \pm 0.21$	$0.24 \times 10^6 \pm 0.05$	0.039
	Jetein	$5.71 \times 10^5 \pm 0.65$	$2.82 \times 10^5 \pm 0.73$	0.048
	1,2-dilinoleoyl-sn-glycerol	$4.43 \times 10^5 \pm 0.62$	$1.54 \times 10^5 \pm 0.41$	0.039
	disepalin	$4.04 \times 10^5 \pm 0.95$	$1.09 \times 10^5 \pm 0.26$	0.05
	12-hydroxy-9,10-dihydrojasmonic acid	$3.10 \times 10^5 \pm 0.24$	$1.94 \times 10^5 \pm 0.16$	0.041
	tonkinelin	$2.56 \times 10^5 \pm 0.34$	$1.22 \times 10^5 \pm 0.29$	0.048
	hydroxyphthioceranic acid	$1.76 \times 10^5 \pm 0.16$	$0.91 \times 10^5 \pm 0.19$	0.036
	cucurbic acid	$1.60 \times 10^5 \pm 0.18$	$0.90 \times 10^5 \pm 0.09$	0.05
	isoketocamphoric acid	$1.23 \times 10^5 \pm 0.12$	$0.49 \times 10^5 \pm 0.14$	0.041
	1-acetoxy-2-hydroxy-5,12,15-heneicosatrien-4-one	$0.98 \times 10^5 \pm 0.11$	$0.39 \times 10^5 \pm 0.07$	0.036
	type IV cyanolipid 20:0 ester	$0.85 \times 10^5 \pm 0.04$	$0.60 \times 10^5 \pm 0.06$	0.041
	prostaglandin D2-1-glyceryl ester	$0.79 \times 10^5 \pm 0.08$	$0.37 \times 10^5 \pm 0.05$	0.036
	(E,E,E)-N-(2-methylpropyl)hexadeca-2,6,8-trien-10-ynamide	$0.46 \times 10^5 \pm 0.05$	$0.23 \times 10^5 \pm 0.02$	0.039
	methyl 8-[3,5-epidioxy-2-(3-hydroperoxy-1-pentenyl)-cyclopentyl]-octanoate	$0.44 \times 10^5 \pm 0.02$	$0.26 \times 10^5 \pm 0.03$	0.036
	citramalic acid	$0.09 \times 10^5 \pm 0.001$	$0.03 \times 10^5 \pm 0.003$	0.036
Phospholipids	PS(31:0)	$3.94 \times 10^6 \pm 0.21$	$2.52 \times 10^6 \pm 0.27$	0.041
	PC(34:1)	$1.47 \times 10^6 \pm 0.21$	$0.84 \times 10^6 \pm 0.08$	0.039
	PC(36:2)	$0.96 \times 10^6 \pm 0.12$	$0.47 \times 10^6 \pm 0.07$	0.039
	PC(36:3)	$0.68 \times 10^6 \pm 0.07$	$0.34 \times 10^6 \pm 0.06$	0.039
	PS(36:4)	$0.35 \times 10^6 \pm 0.01$	$0.20 \times 10^6 \pm 0.02$	0.036
	PC(25:0)	$0.33 \times 10^6 \pm 0.03$	$0.20 \times 10^6 \pm 0.02$	0.048
	PC(36:1)	$0.30 \times 10^6 \pm 0.04$	$0.16 \times 10^6 \pm 0.02$	0.036
	PS(29:0)	$0.17 \times 10^6 \pm 0.02$	$0.09 \times 10^6 \pm 0.01$	0.036
	PE(38:1)	$0.10 \times 10^6 \pm 0.01$	$0.05 \times 10^6 \pm 0.004$	0.036
	1-archaetidyl-D-myo-inositol	$0.07 \times 10^6 \pm 0.005$	$0.04 \times 10^6 \pm 0.007$	0.039
	PS(37:3)	$0.06 \times 10^6 \pm 0.003$	$0.04 \times 10^6 \pm 0.004$	0.036
	PA(32:2)	$0.05 \times 10^6 \pm 0.01$	$0.01 \times 10^6 \pm 0.004$	0.039
	PS(30:1)	$0.05 \times 10^6 \pm 0.004$	$0.02 \times 10^6 \pm 0.003$	0.036
	PS(32:1)	$0.04 \times 10^6 \pm 0.007$	$0.01 \times 10^6 \pm 0.002$	0.039
	PE(38:4)	$0.04 \times 10^6 \pm 0.003$	$0.02 \times 10^6 \pm 0.004$	0.048
	PE(38:4)	$0.03 \times 10^6 \pm 0.004$	$0.02 \times 10^6 \pm 0.002$	0.041
	LPC(18:0)	$0.03 \times 10^6 \pm 0.004$	$0.01 \times 10^6 \pm 0.003$	0.048
	LPE(16:0)	$0.03 \times 10^6 \pm 0.001$	$0.02 \times 10^6 \pm 0.003$	0.039
	PS(29:0)	$0.03 \times 10^6 \pm 0.002$	$0.01 \times 10^6 \pm 0.003$	0.041
	PA(44:3)	$0.016 \times 10^6 \pm 0.003$	$0.005 \times 10^6 \pm 0.002$	0.041
	PA(46:4)	$0.008 \times 10^6 \pm 0.002$	$0.0008 \times 10^6 \pm 0.0001$	0.048
	PI(35:2)	$0.005 \times 10^6 \pm 0.0003$	$0.003 \times 10^6 \pm 0.0006$	0.048

Prenol lipids	geranylcitronellol	$6.22 \times 10^6 \pm 0.28$	$3.97 \times 10^6 \pm 0.68$	0.041
	3-(all-trans-npnaprenyl)benzene-1,2-diol	$1.14 \times 10^6 \pm 0.15$	$0.53 \times 10^6 \pm 0.09$	0.039
	menaquinol-11	$1.09 \times 10^6 \pm 0.12$	$0.57 \times 10^6 \pm 0.11$	0.048
	plastochromanol 8	$0.66 \times 10^6 \pm 0.08$	$0.33 \times 10^6 \pm 0.08$	0.039
	3-demethylubiquinol-10	$0.36 \times 10^6 \pm 0.05$	$0.14 \times 10^6 \pm 0.03$	0.045
	theasapogenol A	$0.27 \times 10^6 \pm 0.04$	$0.10 \times 10^6 \pm 0.01$	0.036
	14-deacetylnudicauline	$0.19 \times 10^6 \pm 0.03$	$0.05 \times 10^6 \pm 0.01$	0.039
	guayulin B	$0.18 \times 10^6 \pm 0.02$	$0.09 \times 10^6 \pm 0.01$	0.039
	Cassaidine	$0.18 \times 10^6 \pm 0.02$	$0.10 \times 10^6 \pm 0.02$	0.048
	reduced coenzyme Q10	$0.13 \times 10^6 \pm 0.01$	$0.06 \times 10^6 \pm 0.02$	0.048
	glisoprenin D	$0.12 \times 10^6 \pm 0.01$	$0.06 \times 10^6 \pm 0.01$	0.036
	3-epipapyriferic acid	$0.12 \times 10^6 \pm 0.02$	$0.05 \times 10^6 \pm 0.004$	0.048
	aplidiasphingosine	$0.11 \times 10^6 \pm 0.007$	$0.06 \times 10^6 \pm 0.007$	0.041
	solavetivone	$0.08 \times 10^6 \pm 0.01$	$0.03 \times 10^6 \pm 0.004$	0.044
	avadharidine	$0.03 \times 10^6 \pm 0.005$	$0.007 \times 10^6 \pm 0.001$	0.05
Sphingolipids	N-triacontanoyphytosphingosine	$7.80 \times 10^4 \pm 1.47$	$3.10 \times 10^4 \pm 0.66$	0.048
	SM(38:1)	$5.05 \times 10^4 \pm 0.63$	$3.72 \times 10^4 \pm 0.53$	0.049
	ganglioside GA2	$1.53 \times 10^4 \pm 0.13$	$1.07 \times 10^4 \pm 0.14$	0.041
Steroids	goyaglycoside c	$4.88 \times 10^6 \pm 0.21$	$3.18 \times 10^6 \pm 0.33$	0.039
	17-oxocycloprotobuxine	$2.89 \times 10^6 \pm 0.27$	$1.60 \times 10^6 \pm 0.22$	0.048
	ACGal C6	$0.12 \times 10^6 \pm 0.02$	$0.036 \times 10^6 \pm 0.006$	0.036
	momordicoside G	$0.12 \times 10^6 \pm 0.02$	$0.036 \times 10^6 \pm 0.005$	0.039
	sitoindoside I	$0.07 \times 10^6 \pm 0.006$	$0.033 \times 10^6 \pm 0.008$	0.041
	testosterone enanthate	$0.05 \times 10^6 \pm 0.002$	$0.033 \times 10^6 \pm 0.004$	0.036
	sulfolithocholic acid	$0.016 \times 10^6 \pm 0.001$	$0.009 \times 10^6 \pm 0.001$	0.041
