# Supplementary material for: Oocyte metabolic function, lipid composition, and developmental potential are altered by diet in older mares
Source: Reproduction. 2022 Jan 28;163(4):183–98. doi: 10.1530/REP-21-0351 (PMC8942336; doi:10.1530/REP-21-0351)
Supplement: Supplementary Table 2. Abundance of oocyte metabolites that were affected by supplementation with RSS2. Single oocytes were analyzed from the same mares Pre and Post approximately two months of supplementation. Results are presented as mean ± SEM. [file supplementary_table_2.pdf]

Supplementary Table 2. Abundance of oocyte metabolites that were affected by supplementation with RSS2. Single oocytes were analyzed from the same mares Pre and Post approximately two months of supplementation. Results are presented as mean ± SEM.

Superclass	Metabolite	Pre	Post	P value
Alkaloids and derivatives	Pseudoconhydrine	$4.14 \times 10^4 \pm 0.30$	$2.66 \times 10^4 \pm 0.24$	0.022
	13alpha-(caproyloxy)lupanine	$3.71 \times 10^3 \pm 0.64$	$1.01 \times 10^3 \pm 0.27$	0.022
	17-O-acetyltetraphyllicine	$2.12 \times 10^4 \pm 0.43$	$0.37 \times 10^4 \pm 0.25$	0.034
	Jafrine	$3.70 \times 10^3 \pm 0.75$	$1.06 \times 10^3 \pm 0.13$	0.035
	Ecgonine methyl ester	$1.49 \times 10^5 \pm 0.30$	$0.42 \times 10^5 \pm 0.13$	0.042
	Strictosidine aglycone	$4.45 \times 10^4 \pm 0.54$	$2.52 \times 10^4 \pm 0.43$	0.064
	Prosopinine	$8.67 \times 10^3 \pm 0.85$	$5.84 \times 10^3 \pm 0.74$	0.09
	Tropinone	$1.05 \times 10^4 \pm 0.23$	$0.40 \times 10^4 \pm 0.13$	0.093
Benzenoids	Aristolochic acid III methyl ester	$1.37 \times 10^4 \pm 0.11$	$0.51 \times 10^4 \pm 0.07$	0.003
	6-[oxo-[2-[oxo-2-phenylethylamino)methyl]anilino] methyl]-1-cyclohex-3-enecarboxylis acid	$6.45 \times 10^2 \pm 0.16$	$3.89 \times 10^2 \pm 0.44$	0.009
	Methyl benzoate	$3.96 \times 10^3 \pm 0.31$	$2.10 \times 10^3 \pm 0.21$	0.012
	Grenadamide	$7.27 \times 10^4 \pm 0.89$	$2.95 \times 10^4 \pm 0.35$	0.016
	Fenpiprane	$2.23 \times 10^4 \pm 0.52$	$0.25 \times 10^4 \pm 0.05$	0.025
	Carbuterol	$1.14 \times 10^4 \pm 0.24$	$0.24 \times 10^6 \pm 0.06$	0.031
	(2S)-2-[[[4-[[4-[[[(2-methylpropan-2-yl)oxy-oxomethyl]amino]methyl]anilino]-oxomethyl]-1H-imidazol-5-yl]-oxomethyl]amino]propanoic acid tert-butyl ester	$1.96 \times 10^4 \pm 0.52$	$0.10 \times 10^4 \pm 0.05$	0.032
	Alprenolol	$1.55 \times 10^5 \pm 0.11$	$0.99 \times 10^5 \pm 0.13$	0.038
	Oxybuprocaine	$2.04 \times 10^4 \pm 0.30$	$4.91 \times 10^4 \pm 0.81$	0.040
	4-Allyl-2,6-dimethoxyphenol	$5.42 \times 10^4 \pm 0.21$	$3.98 \times 10^4 \pm 0.40$	0.044
	N-[3-(4-morpholinylsulfonyl)phenyl]-4-(2-oxo-1-pyrrolidinyl)benzamide	$0.68 \times 10^4 \pm 0.49$	$6.97 \times 10^4 \pm 1.95$	0.046
	Atenolol	$2.34 \times 10^5 \pm 0.16$	$1.58 \times 10^5 \pm 0.18$	0.048
	1-(3,5-dimethyl-4-isoxazolyl)-3-[(2S,3S)-5-[(2S)-1-hydroxypropan-2-yl]-2-[[(4-methoxyphenyl)methyl-methylamino]methyl]-3-methyl-6-oxo-3,4-dihydro-2H-1,5-benzoxazocin-8-yl]urea	$2.90 \times 10^4 \pm 0.76$	$0.62 \times 10^4 \pm 0.14$	0.056
	Aristolochic acid	$6.33 \times 10^3 \pm 0.11$	$2.92 \times 10^3 \pm 0.38$	0.062
	2-({[(4-methoxyphenyl)methyl](methyl)amino}methyl)-2-methylpropane-1,3-diol_2	$1.39 \times 10^4 \pm 0.47$	$0.16 \times 10^4 \pm 0.03$	0.080
	6-amino-4-(2-methoxyphenyl)-3-propyl-2,4-dihydropyrano[2,3-c]pyrazole-5-carbonitrile	$5.05 \times 10^2 \pm 0.47$	$3.24 \times 10^2 \pm 0.53$	0.088
	trans-1-Phenyl-1-pentene	$2.20 \times 10^5 \pm 0.27$	$1.35 \times 10^5 \pm 0.20$	0.090
Lignans	Myricatomentoside I	$4.86 \times 10^4 \pm 0.13$	$3.09 \times 10^4 \pm 0.43$	0.021
Nucleosides	dTDP-5-dimethyl-L-lyxose	$4.68 \times 10^4 \pm 0.28$	$2.05 \times 10^4 \pm 0.21$	0.0007
Organic acids	Lotusanine B	$3.93 \times 10^5 \pm 0.26$	$1.56 \times 10^5 \pm 0.28$	0.003

	Thiazinotrienomycin G	$2.24 \times 10^4 \pm 0.01$	$1.51 \times 10^4 \pm 0.13$	0.016
	(2S)-2-[[(2R)-2-[(1S)-1-hydroxy-2-(hydroxyamino)-2-oxoethyl]-4-methyl-1-oxopentyl]amino]-2-phenylacetic acid cyclopentyl ester	$2.11 \times 10^4 \pm 0.01$	$1.37 \times 10^4 \pm 0.13$	0.016
	N2-Acetyl-L-aminoadipyl-delta-phosphate	$1.87 \times 10^4 \pm 0.13$	$1.09 \times 10^4 \pm 0.13$	0.019
	Restricticin	$2.34 \times 10^4 \pm 0.27$	$1.14 \times 10^4 \pm 0.12$	0.020
	D-Chicoric acid	$0.43 \times 10^4 \pm 0.16$	$2.12 \times 10^4 \pm 0.40$	0.022
	Haligramide B	$9.46 \times 10^3 \pm 0.51$	$6.77 \times 10^3 \pm 0.59$	0.036
	6-octenoylglycine	$1.22 \times 10^4 \pm 0.24$	$0.39 \times 10^4 \pm 0.06$	0.037
	Melagatran	$2.93 \times 10^4 \pm 0.84$	$0.16 \times 10^4 \pm 0.04$	0.040
	Ichthyotherminolide	$6.89 \times 10^4 \pm 1.75$	$1.14 \times 10^4 \pm 0.24$	0.041
	N-(2-Hydroxyethyl)decanamide	$1.17 \times 10^4 \pm 0.29$	$0.25 \times 10^4 \pm 0.05$	0.047
	Glutathione	$0.23 \times 10^5 \pm 0.15$	$1.14 \times 10^5 \pm 0.25$	0.048
	Ceanothine C	$1.90 \times 10^4 \pm 0.37$	$0.73 \times 10^4 \pm 0.11$	0.049
	Azotochelin	$0.82 \times 10^7 \pm 0.65$	$3.92 \times 10^7 \pm 0.79$	0.050
	GABA-stearamide	$3.05 \times 10^5 \pm 0.39$	$1.62 \times 10^5 \pm 0.27$	0.051
	N-decanoylglycine	$4.72 \times 10^4 \pm 0.31$	$3.24 \times 10^4 \pm 0.38$	0.053
	N-Butylacetamide	$5.60 \times 10^2 \pm 0.54$	$3.48 \times 10^2 \pm 0.47$	0.056
	3-(3,4-dimethoxyphenyl)-N-{2-[4-methoxy-3-(sulfooxy)phenyl]ethyl}prop-2-enimidic acid	$2.09 \times 10^6 \pm 1.11$	$7.35 \times 10^6 \pm 0.14$	0.057
	Cyclosquamosin G	$6.17 \times 10^3 \pm 1.53$	$1.56 \times 10^3 \pm 0.37$	0.057
	DES(2-methylbutanoyl)pravastatin	$1.10 \times 10^5 \pm 0.10$	$0.71 \times 10^5 \pm 0.10$	0.062
	3,12-dihydroxylaurate	$2.16 \times 10^5 \pm 0.30$	$1.17 \times 10^5 \pm 0.17$	0.063
	Sorbitan tristearate	$1.09 \times 10^5 \pm 0.10$	$0.60 \times 10^5 \pm 0.14$	0.064
	Melagatran	$2.14 \times 10^6 \pm 0.73$	$0.12 \times 10^6 \pm 0.04$	0.067
	D-2-Hydroxyglutaric acid	$2.45 \times 10^5 \pm 0.20$	$1.60 \times 10^5 \pm 0.24$	0.074
	Gabapentin enacarbil	$2.29 \times 10^4 \pm 0.82$	$0.10 \times 10^4 \pm 0.01$	0.077
	L-Theanine	$1.35 \times 10^5 \pm 0.07$	$1.05 \times 10^5 \pm 0.09$	0.077
	Leucylproline	$6.46 \times 10^3 \pm 1.64$	$2.07 \times 10^3 \pm 0.33$	0.079
	Tributyl phosphate	$5.31 \times 10^4 \pm 0.54$	$3.61 \times 10^4 \pm 0.39$	0.085
	methotrexate	$7.37 \times 10^3 \pm 0.55$	$4.85 \times 10^3 \pm 0.83$	0.088
	2-(3'-Methylthio)propylmalic acid	$1.79 \times 10^5 \pm 0.19$	$1.23 \times 10^5 \pm 0.13$	0.093
	Phaseolic acid	$8.04 \times 10^4 \pm 0.95$	$4.38 \times 10^4 \pm 1.13$	0.093
	(Z)-N-hydroxy-11-methyldodec-2-enamide	$2.77 \times 10^6 \pm 0.69$	$0.99 \times 10^6 \pm 0.19$	0.093
	Hexacosanoic acid isobutylamide	$5.29 \times 10^3 \pm 0.70$	$3.11 \times 10^3 \pm 0.52$	0.093
	GABA-stearamide	$3.57 \times 10^4 \pm 0.81$	$1.50 \times 10^4 \pm 0.25$	0.098
Organic nitrogen compounds	Linoleoyl ethanolamide	$3.19 \times 10^4 \pm 0.32$	$0.98 \times 10^4 \pm 0.17$	0.004
	1-hexadecyl-2-ammonio-2-deoxy-sn-glycerol	$1.20 \times 10^5 \pm 0.28$	$0.75 \times 10^5 \pm 0.08$	0.010
	15-methylhexadecasphinganine	$6.74 \times 10^5 \pm 0.27$	$4.32 \times 10^5 \pm 0.41$	0.013
	N-(2-hydroxyheptacosanoyl)-4-hydroxy-15-methylhexadecasphinganine-1-phosphocholine	$4.80 \times 10^5 \pm 0.61$	$1.94 \times 10^5 \pm 0.48$	0.028
	N-icosanoyl-4-hydroxy-15-methylhexadecasphinganine-1-phosphocholine	$1.48 \times 10^5 \pm 0.13$	$0.77 \times 10^5 \pm 0.17$	0.037
	n-oleoylethanolamine	$1.59 \times 10^5 \pm 0.17$	$0.93 \times 10^5 \pm 0.12$	0.045

	Dioctylamine	$5.44 \times 10^5 \pm 0.45$	$3.58 \times 10^5 \pm 0.40$	0.047
	Halaminol A	$1.10 \times 10^5 \pm 0.09$	$0.70 \times 10^5 \pm 0.09$	0.047
	Octamylamine	$6.73 \times 10^4 \pm 0.66$	$4.08 \times 10^4 \pm 0.59$	0.053
	Octylamine	$4.71 \times 10^5 \pm 0.77$	$2.39 \times 10^5 \pm 0.26$	0.062
	N-gondoylethanolamine	$2.13 \times 10^6 \pm 0.32$	$1.12 \times 10^6 \pm 0.16$	0.062
	N-(2-hydroxypentacosanoyl)-15-methylhexadecasphing-4-enine-1-phosphocholine	$1.47 \times 10^5 \pm 0.20$	$0.77 \times 10^5 \pm 0.14$	0.063
	N-(11Z,14Z)-eicosadienoylethanolamine	$3.14 \times 10^5 \pm 0.36$	$1.80 \times 10^5 \pm 0.31$	0.063
	2-(5-Methyl-2-furanyl)-3-piperidinol	$1.07 \times 10^4 \pm 0.19$	$0.49 \times 10^4 \pm 0.07$	0.066
	Octodrine	$9.92 \times 10^4 \pm 3.21$	$1.48 \times 10^4 \pm 1.01$	0.090
Organic oxygen compounds	2-[(2R,4aR,12aS)-5-methyl-6-oxo-8-[[oxo-(propan-2-ylamino)methyl]amino]-2,3,4,4a,12,12a-hexahydropyrano[2,3-c][1,5]benzoxazocin-2-yl]-N-propylacetamide	$5.26 \times 10^4 \pm 0.35$	$1.61 \times 10^4 \pm 0.25$	0.0005
	Dimethyl 2-galloylgalactarate	$9.67 \times 10^4 \pm 0.73$	$3.03 \times 10^4 \pm 0.37$	0.0005
	Fluvirucin B2	$4.49 \times 10^4 \pm 0.23$	$2.79 \times 10^4 \pm 0.26$	0.013
	Pentaethylene glycol monododecyl ether	$1.54 \times 10^5 \pm 0.06$	$0.99 \times 10^5 \pm 0.10$	0.015
	Fortimicin KL1	$4.95 \times 10^3 \pm 0.54$	$1.85 \times 10^3 \pm 0.47$	0.016
	6-[1-carboxy-2-(4-methoxy-1-benzofuran-5-yl)-2-oxoethyl]-3,4,5-trihydroxyoxane-2-carboxylic acid	$2.51 \times 10^5 \pm 0.22$	$1.22 \times 10^5 \pm 0.21$	0.018
	Alpha-Butyl-omega-hydroxypoly(oxyethylene) poly(oxypropylene)	$5.98 \times 10^4 \pm 0.56$	$3.26 \times 10^4 \pm 0.31$	0.018
	Midecamycin acetate	$6.37 \times 10^2 \pm 0.46$	$3.30 \times 10^2 \pm 0.58$	0.020
	Itoside K	$5.46 \times 10^4 \pm 0.67$	$2.44 \times 10^4 \pm 0.26$	0.020
	6-[5-(1-carboxyethyl)-4-hydroxy-2-methoxyphenoxy]-3,4,5-trihydroxyoxane-2-carboxylic acid	$4.85 \times 10^5 \pm 0.32$	$2.84 \times 10^5 \pm 0.39$	0.021
	Picrocrocin	$7.47 \times 10^5 \pm 1.22$	$2.37 \times 10^5 \pm 0.71$	0.030
	5-decanoyl-2-nonylpyridine	$3.79 \times 10^4 \pm 0.26$	$2.37 \times 10^4 \pm 0.32$	0.036
	N'-monoacetylchitobiose-6'-phosphate(1-)	$1.13 \times 10^5 \pm 0.12$	$0.65 \times 10^5 \pm 0.08$	0.041
	Ciramadol	$2.19 \times 10^6 \pm 0.28$	$1.13 \times 10^6 \pm 0.16$	0.041
	N-[(2R,3R,6R)-2-(hydroxymethyl)-6-[2-oxo-2-[2-(1-piperidinyl)ethylamino]ethyl]-3-oxanyl]propanamide	$1.46 \times 10^4 \pm 0.28$	$2.79 \times 10^4 \pm 0.32$	0.045
	N-(2-hydroxyheptacosanoyl)-15-methylhexadecasphing-4-enine	$1.28 \times 10^5 \pm 0.20$	$0.60 \times 10^5 \pm 0.10$	0.054
	3,6,9,12,15-Pentaoxaheptadecane	$1.02 \times 10^5 \pm 0.17$	$0.65 \times 10^5 \pm 0.06$	0.062
	8,8-Diethoxy-2,6-dimethyl-2-octanol	$2.77 \times 10^4 \pm 0.15$	$1.93 \times 10^4 \pm 0.25$	0.062
	(N-acetylneuraminosyl(a2-6)lactosamine)	$6.27 \times 10^5 \pm 0.34$	$4.59 \times 10^5 \pm 0.49$	0.064
	Scabran G3	$7.21 \times 10^5 \pm 0.47$	$5.40 \times 10^5 \pm 0.47$	0.069
	N,O-Didesmethylvenlafaxine	$2.06 \times 10^5 \pm 0.18$	$1.34 \times 10^5 \pm 0.20$	0.072
	4-hydroxybenzaldehyde	$2.12 \times 10^4 \pm 0.21$	$1.44 \times 10^4 \pm 0.15$	0.076
	Sorgoleone 358	$1.83 \times 10^4 \pm 0.17$	$1.21 \times 10^4 \pm 0.17$	0.082
	Glucocamelinin	$5.26 \times 10^3 \pm 1.02$	$2.43 \times 10^3 \pm 0.45$	0.088

	(2E,4E)-Octa-2,4-dienal	$9.72 \times 10^{2} \pm 1.03$	$6.30 \times 10^{2} \pm 0.95$	0.097
	2,4-Dihydroxyacetophenone 5-sulfate	$3.69 \times 10^{3} \pm 1.00$	$1.24 \times 10^{3} \pm 0.11$	0.098
	N-octacosanoyl-4-hydroxy-15-methylhexadecasphinganine	$4.85 \times 10^{4} \pm 0.91$	$2.30 \times 10^{4} \pm 0.52$	0.099
	N-(2-hydroxytetracosanoyl)-15-methylhexadecasphing-4-enine	$6.82 \times 10^{4} \pm 1.37$	$3.16 \times 10^{4} \pm 0.70$	0.099
Organoheterocyclic compounds	trans-2-Butyl-5-pentylpyrrolidine	$6.04 \times 10^{4} \pm 0.75$	$2.30 \times 10^{4} \pm 0.26$	0.014
	5,8-Dihydro-6-(4-methyl-3-pentenyl)-1,2,3,4-tetrathiocin	$3.13 \times 10^{4} \pm 0.28$	$1.45 \times 10^{4} \pm 0.25$	0.016
	2,5-dimethylfuran	$1.09 \times 10^{3} \pm 0.10$	$0.53 \times 10^{3} \pm 0.07$	0.016
	Austinol	$3.49 \times 10^{4} \pm 0.14$	$2.26 \times 10^{4} \pm 0.26$	0.018
	2-carboxy-5,7-dimethyl-4-octanolide	$1.60 \times 10^{5} \pm 0.18$	$0.86 \times 10^{5} \pm 0.05$	0.021
	5-(1-methyl-2-benzimidazolyl)-2-thiophenecarboxaldehyde	$2.23 \times 10^{4} \pm 0.34$	$0.91 \times 10^{4} \pm 0.07$	0.025
	Arachidonoylmorpholine	$3.67 \times 10^{4} \pm 0.28$	$2.19 \times 10^{4} \pm 0.31$	0.030
	2-isopropyl-5-methylfuran	$7.71 \times 10^{3} \pm 0.71$	$4.25 \times 10^{3} \pm 0.65$	0.030
	Iproniazid	$1.62 \times 10^{3} \pm 0.10$	$1.12 \times 10^{3} \pm 0.10$	0.036
	(6R,7S,8aS)-N-(5-aminopentyl)-6-(4-hydroxyphenyl)-1,4-dioxo-2,3,6,7,8,8a-hexahydropyrrolo[1,2-a]pyrazine-7-carboxamide	$2.01 \times 10^{5} \pm 0.18$	$1.28 \times 10^{5} \pm 0.12$	0.037
	2-hexyl-4-methyl-5-ethyloxazole	$4.42 \times 10^{3} \pm 0.88$	$1.23 \times 10^{3} \pm 0.41$	0.041
	Famotidine	$9.80 \times 10^{4} \pm 0.69$	$6.29 \times 10^{4} \pm 0.84$	0.043
	1-hexadecanoylpyrrolidine	$5.60 \times 10^{4} \pm 0.55$	$3.31 \times 10^{4} \pm 0.47$	0.045
	2-cyclopropyl-1-[(2S,3R)-2-(hydroxymethyl)-3-phenyl-6-(3-pyridinylmethyl)-1,6-diazaspiro[3.3]heptan-1-yl]ethanone	$2.15 \times 10^{4} \pm 0.19$	$1.20 \times 10^{4} \pm 0.24$	0.047
	4-methoxy-N-[2-[(4-nitrophenyl)methylthio]-4-oxo-3-quinazolinyl]benzamide	$0.35 \times 10^{6} \pm 0.23$	$2.76 \times 10^{6} \pm 0.74$	0.047
	Cetilistat	$6.74 \times 10^{5} \pm 0.60$	$3.99 \times 10^{5} \pm 0.66$	0.047
	4-[(3aR,4R,9bR)-1-[cyclohexyl(oxo)methyl]-4-(hydroxymethyl)-2,3,3a,4,5,9b-hexahydropyrrolo[3,2-c]quinolin-8-yl]benzonitrile	$1.04 \times 10^{5} \pm 0.27$	$0.19 \times 10^{5} \pm 0.07$	0.049
	1-butyl-5-[1-[2-(1H-indol-3-yl)ethylamino]ethylidene]-1,3-diazinane-2,4,6-trione	$2.53 \times 10^{3} \pm 0.37$	$1.17 \times 10^{3} \pm 0.26$	0.051
	Pyrinodemin D	$1.11 \times 10^{5} \pm 0.08$	$0.76 \times 10^{5} \pm 0.09$	0.052
	Brassicanal B	$0.38 \times 10^{6} \pm 0.22$	$1.78 \times 10^{6} \pm 0.43$	0.060
	7-[2-hydroxy-3-(4-methoxyphenoxy)propyl]-1,3-dimethyl-8-(1-piperidinyl)purine-2,6-dione	$4.67 \times 10^{4} \pm 1.61$	$0.10 \times 10^{4} \pm 0.04$	0.062
	Ditalimfos	$0.79 \times 10^{5} \pm 0.44$	$3.37 \times 10^{5} \pm 0.80$	0.063
	Phendimetrazine	$7.26 \times 10^{4} \pm 0.53$	$5.20 \times 10^{4} \pm 0.51$	0.064
	Brachyamide B	$1.73 \times 10^{4} \pm 0.38$	$0.61 \times 10^{4} \pm 0.16$	0.071
	1,2,3,4,5,6-hexahydro-5-(1-hydroxyethylidene)-7H-cyclopenta[b]pyridin-7-one	$1.20 \times 10^{4} \pm 0.28$	$0.33 \times 10^{4} \pm 0.15$	0.072
	1-ethylpiperidine	$2.16 \times 10^{3} \pm 0.48$	$0.65 \times 10^{3} \pm 0.30$	0.073
	6,7-Epoxy-3Z,9Z-tricosadiene	$4.73 \times 10^{5} \pm 0.37$	$2.95 \times 10^{5} \pm 0.55$	0.075
	Indole-3-carbinol	$6.00 \times 10^{3} \pm 1.43$	$2.11 \times 10^{3} \pm 0.32$	0.077

	Scorodocarpine C	2.73 x 10^4 ± 0.32	1.76 x 10^4 ± 0.18	0.077
	Hapovine	1.78 x 10^3 ± 0.26	0.93 x 10^3 ± 0.20	0.079
	3-Isopropyl-2-methoxy-5-methylpyrazine	9.91 x 10^4 ± 1.35	5.77 x 10^4 ± 0.87	0.084
	Azepan-2-one	1.03 x 10^6 ± 0.24	0.40 x 10^6 ± 0.05	0.085
	N-[(Z)-dodec-2-enoyl]morpholine	3.96 x 10^3 ± 1.12	1.00 x 10^3 ± 0.32	0.088
	Phomacin B	2.11 x 10^4 ± 0.24	1.33 x 10^4 ± 0.21	0.093
	2-[[3-(4-morpholinylmethyl)-2H-1-benzopyran-8-yl]oxymethyl]morpholine	4.73 x 10^3 ± 1.30	1.39 x 10^3 ± 0.37	0.094
	Pipercallosidine	2.33 x 10^3 ± 0.61	0.77 x 10^3 ± 0.20	0.097
	Sieboldine A	1.17 x 10^6 ± 0.14	0.71 x 10^6 ± 0.12	0.099
Phenylpropanoids and polyketides	3-(2,4-dihydroxy-3,5-dimethoxyphenyl)prop-2-enoic acid	1.21 x 10^5 ± 0.10	0.41 x 10^5 ± 0.05	0.0008
	Cytotrienin A	3.21 x 10^5 ± 0.32	0.52 x 10^4 ± 0.16	0.004
	Feruloylagmatine	2.13 x 10^4 ± 0.25	1.22 x 10^4 ± 0.19	0.009
	1,2,6-trigalloyl-beta-D-glucopyranose	3.06 x 10^4 ± 0.19	1.51 x 10^4 ± 0.24	0.011
	{5-[2,3-dioxo-3-(2,4,6-trihydroxy-3-methoxyphenyl)propyl]-2-hydroxyphenyl}oxidanesulfonic acid	8.67 x 10^3 ± 1.43	1.75 x 10^3 ± 0.51	0.015
	Pinocembrin 7-O-neohesperidoside 6''-O-acetate	3.03 x 10^5 ± 0.17	1.65 x 10^5 ± 0.25	0.015
	8-hydroxyluteolin 7-[6'''-acetylallosyl-(1->2)-glucoside]	2.10 x 10^4 ± 0.12	1.35 x 10^4 ± 0.13	0.018
	4-[1-ethyl-2-(4-methylphenyl)butyl]phenol	2.29 x 10^3 ± 0.43	0.52 x 10^3 ± 0.14	0.021
	3-[3,4-dihydroxy-5-(3,4,5-trihydroxybenzoyloxy)benzoyloxy]-5-hydroxy-4-methoxybenzoic acid	3.91 x 10^4 ± 0.42	2.04 x 10^4 ± 0.21	0.021
	[10-butanoyl-5-hydroxy-6-(2-hydroxypropyl)-2,2-dimethyl-8-oxo-2H,3H,4H,8H-pyrano[3,2-g]chromen-3-yl]oxidanesulfonic acid	3.32 x 10^3 ± 0.27	1.90 x 10^3 ± 0.24	0.022
	N-[[(8R,9R)-6-[(2S)-1-hydroxypropan-2-yl]-8-methyl-5-oxo-10-oxa-1,6,14,15-tetrazabicyclo[10.3.0]pentadeca-12,14-dien-9-yl]methyl]-N-methylcyclopropanecarboxamide	2.76 x 10^5 ± 0.72	0.07 x 10^5 ± 0.02	0.027
	N-[(3R,9S,10S)-12-[(2R)-1-hydroxypropan-2-yl]-3,10-dimethyl-9-[[methyl(propyl)amino]methyl]-13-oxo-2,8-dioxa-12-azabicyclo[12.4.0]octadeca-1(14),15,17-trien-16-yl]-4-pyridinecarboxamide	1.75 x 10^5 ± 0.37	0.39 x 10^5 ± 0.06	0.030
	2-hydroxy-24-keto-octacosanolide	2.35 x 10^5 ± 0.18	1.50 x 10^5 ± 0.15	0.031
	3-[4,10'-epoxylinalyl]-5-methyl coumarin	1.15 x 10^4 ± 0.21	0.32 x 10^4 ± 0.12	0.036
	5-hydroxy-6-methoxycoumarin 7-glucoside	1.09 x 10^5 ± 0.10	0.68 x 10^5 ± 0.06	0.036
	Rhamnetin	1.91 x 10^5 ± 0.34	0.76 x 10^5 ± 0.07	0.041
	(3S,3'S,4'R,6'S,8'R,8'aR)-5-[3-(carbamoylamino)prop-1-ynyl]-6'-[4-(2-hydroxyethoxy)phenyl]-1',2-dioxo-3',4'-diphenyl-N-prop-2-enyl-8'-	5.06 x 10^3 ± 0.33	3.45 x 10^3 ± 0.36	0.041

spiro[1H-indole-3,7'-4,6,8,8a-tetrahydro-3H-pyrrolo[2,1-c][1,4]oxazine]carboxamide			
2-cinnamoyl-1,6-digalloyl-beta-D-glucopyranose	$1.25 \times 10^5 \pm 0.13$	$0.75 \times 10^5 \pm 0.09$	0.045
Kadsuphilol F	$1.15 \times 10^5 \pm 0.09$	$0.78 \times 10^5 \pm 0.08$	0.047
Acacetin 7-(4'''-acetylrutinoside)	$1.90 \times 10^4 \pm 0.19$	$1.20 \times 10^4 \pm 0.12$	0.047
Chakaflavonoside A	$1.22 \times 10^4 \pm 0.11$	$0.81 \times 10^4 \pm 0.81$	0.048
Volkensiflavone	$5.00 \times 10^4 \pm 0.39$	$3.16 \times 10^4 \pm 0.48$	0.052
Epothilone C6	$8.11 \times 10^4 \pm 2.04$	$1.86 \times 10^4 \pm 0.58$	0.055
Persicarin	$8.09 \times 10^4 \pm 2.65$	$0.64 \times 10^4 \pm 0.34$	0.064
2-(3,4-dihydroxyphenyl)-3,5-dihydroxy-7-{[3,4,5-trihydroxy-6-(hydroxymethyl)oxan-2-yl]oxy}-4H-chromen-4-one	$1.30 \times 10^5 \pm 0.14$	$0.73 \times 10^5 \pm 0.15$	0.070
Hesperetin 7-glucoside	$8.34 \times 10^3 \pm 0.73$	$5.45 \times 10^3 \pm 0.81$	0.077
curcumin 4',4''-O-D-digentiobioside	$4.67 \times 10^3 \pm 0.50$	$3.03 \times 10^3 \pm 0.38$	0.079
3',6-disinapoylsucrose	$8.22 \times 10^5 \pm 0.51$	$6.40 \times 10^5 \pm 0.49$	0.084
Rhoifolin	$8.18 \times 10^3 \pm 1.26$	$4.52 \times 10^3 \pm 0.77$	0.084
Palmerolide A	$1.29 \times 10^5 \pm 0.18$	$0.75 \times 10^5 \pm 0.13$	0.098
3-(1,1-dimethylallyl)scopoletin 7-glucoside	$1.85 \times 10^3 \pm 0.34$	$0.93 \times 10^3 \pm 0.16$	0.098
