# Supplementary figures and images for: Oocyte metabolic function, lipid composition, and developmental potential are altered by diet in older mares
Source: Reproduction. 2022 Jan 28;163(4):183–98. doi: 10.1530/REP-21-0351 (PMC8942336; doi:10.1530/REP-21-0351)

(A)

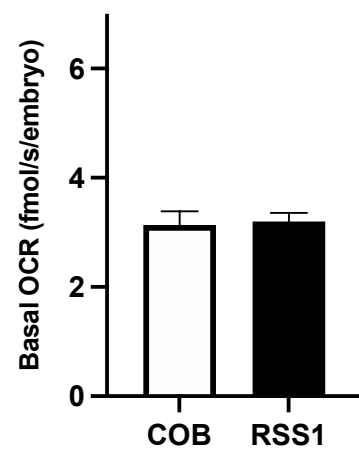

(B)

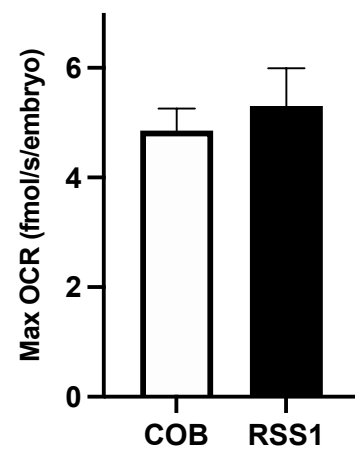

Supplement: Supplementary Figure 1. Aerobic metabolism, based on oxygen consumption rates (OCR), in embryos 2 days after intracytoplasmic sperm injection of oocytes from older mares supplemented with grain and corn oil (COB) or complex nutrients to support health and reproductive function (RSS1). (A) Basal OCR  [file supplementary_figure_1.pdf]
